# Multi-locus genome-wide association study of fusarium head blight in relation to days to anthesis and plant height in a spring wheat association panel

**DOI:** 10.3389/fpls.2023.1166282

**Published:** 2023-06-29

**Authors:** Adrian L. Cabral, Yuefeng Ruan, Richard D. Cuthbert, Lin Li, Wentao Zhang, Kerry Boyle, Samia Berraies, Maria Antonia Henriquez, Andrew Burt, Santosh Kumar, Pierre Fobert, Isabelle Piche, Firdissa E. Bokore, Brad Meyer, Jatinder Sangha, Ron E. Knox

**Affiliations:** ^1^Swift Current Research and Development Centre, Agriculture and Agri-Food Canada, Swift Current, SK, Canada; ^2^Aquatic and Crop Resource Development Research Centre, National Research Council of Canada, Saskatoon, SK, Canada; ^3^Morden Research and Development Centre, Agriculture and Agri-Food Canada, Morden, MB, Canada; ^4^Ottawa Research and Development Centre, Agriculture and Agri-Food Canada, Ottawa, ON, Canada; ^5^Brandon Research and Development Centre, Agriculture and Agri-Food Canada, Brandon, MB, Canada; ^6^Aquatic and Crop Resource Development Research Centre, National Research Council of Canada, Ottawa, ON, Canada

**Keywords:** Fusarium head blight, spring wheat, QTN, GWAS, plant height, days to anthesis

## Abstract

Fusarium head blight (FHB) is a highly destructive fungal disease of wheat to which host resistance is quantitatively inherited and largely influenced by the environment. Resistance to FHB has been associated with taller height and later maturity; however, a further understanding of these relationships is needed. An association mapping panel (AMP) of 192 predominantly Canadian spring wheat was genotyped with the wheat 90K single-nucleotide polymorphism (SNP) array. The AMP was assessed for FHB incidence (INC), severity (SEV) and index (IND), days to anthesis (DTA), and plant height (PLHT) between 2015 and 2017 at three Canadian FHB-inoculated nurseries. Seven multi-environment trial (MET) datasets were deployed in a genome-wide association study (GWAS) using a single-locus mixed linear model (MLM) and a multi-locus random SNP-effect mixed linear model (mrMLM). MLM detected four quantitative trait nucleotides (QTNs) for INC on chromosomes 2D and 3D and for SEV and IND on chromosome 3B. Further, mrMLM identified 291 QTNs: 50 (INC), 72 (SEV), 90 (IND), 41 (DTA), and 38 (PLHT). At two or more environments, 17 QTNs for FHB, DTA, and PLHT were detected. Of these 17, 12 QTNs were pleiotropic for FHB traits, DTA, and PLHT on chromosomes 1A, 1D, 2D, 3B, 5A, 6B, 7A, and 7B; two QTNs for DTA were detected on chromosomes 1B and 7A; and three PLHT QTNs were located on chromosomes 4B and 6B. The 1B DTA QTN and the three pleiotropic QTNs on chromosomes 1A, 3B, and 6B are potentially identical to corresponding quantitative trait loci (QTLs) in durum wheat. Further, the 3B pleiotropic QTN for FHB INC, SEV, and IND co-locates with *TraesCS3B02G024900* within the *Fhb1* region on chromosome 3B and is ~3 Mb from a cloned *Fhb1* candidate gene *TaHRC*. While the PLHT QTN on chromosome 6B is putatively novel, the 1B DTA QTN co-locates with a disease resistance protein located ~10 Mb from a *Flowering Locus T1-like* gene *TaFT3-B1*, and the 7A DTA QTN is ~5 Mb away from a maturity QTL *QMat.dms-7A.3* of another study. GWAS and QTN candidate genes enabled the characterization of FHB resistance in relation to DTA and PLHT. This approach should eventually generate additional and reliable trait-specific markers for breeding selection, in addition to providing useful information for FHB trait discovery.

## Introduction

Bread wheat (*Triticum aestivum* L.) is a hexaploid species (2n = 6x = 42; AABBDD) with a 17-gigabase-pair genome (Brenchley et al., 2012) and accounts for approximately 95% of the wheat grown worldwide (Dubcovsky and Dvorak, 2007; Shewry, 2009). Fusarium head blight (FHB) or scab of wheat is a yield- and quality-limiting disease caused primarily by the fungus *Fusarium graminearum* Schwabe [teleomorph *Gibberella zeae* (Schwein.) Petch]. Globally, FHB ranks second only to leaf rust as a disease responsible for the largest yield losses in wheat (Savary et al., 2019). FHB symptoms include premature bleached spikelets, discolored rachises, and white or pink Fusarium-damaged kernels (FDK), which weigh less than healthy kernels (McCartney et al., 2007). Fusarium-infected kernels contain a mycotoxin deoxynivalenol (DON), which is a contaminant in commercial foods and cattle feed and is toxic to humans and animals (Desjardins and Hohn, 1997; Gilbert and Tekauz, 2000; Fung and Clark, 2004).

Resistance to FHB has been classified into three main types: Type I (resistance to initial infection), Type II (resistance to spread within the spike), and Type III resistance to the accumulation of DON in infected kernels (Schroeder and Christensen, 1963; Mesterhazy, 1995; Parry et al., 1995). FHB severity (SEV) or Type II resistance quantitative trait loci (QTLs) on chromosome arms 3BS and 6BS were detected in the Chinese wheat cultivar Sumai 3 (Waldron et al., 1999; Anderson et al., 2001). Molecular markers for the 3BS QTL designated as *Fhb1* (Bai et al., 1999; Waldron et al., 1999; Anderson et al., 2001; Zhou et al., 2002; Guo et al., 2003; Liu and Anderson, 2003a; Liu and Anderson, 2003b; Yang et al., 2003; Cuthbert et al., 2006; Liu et al., 2006; Pumphrey et al., 2007; Bernardo et al., 2012; Randhawa et al., 2013) and the 6BS QTL or *Fhb2* (Cuthbert et al., 2007; Zhao et al., 2018) have been widely deployed toward the development of new FHB-tolerant cultivars in global wheat breeding programs. Further, *Fhb4* (Xue et al., 2010) and Sumai 3-derived *Fhb5* on chromosome 5AS (Xue et al., 2011) are two of the well-studied loci for FHB incidence (INC) or Type I resistance. Of the above four loci, *Fhb1* is a major, stable locus estimated to confer an average reduction of 27% in FDK of spring wheat (Pumphrey et al., 2007). However, on its own, *Fhb1* does not provide effective resistance to FHB (McMullen et al., 2012). Thus far, two candidate genes at the *Fhb1* locus have been cloned: a pore-forming toxin-like (PFT) gene (Rawat et al., 2016) and a haplotype Clark histidine-rich calcium-binding (*TaHRC*) protein gene (Su et al., 2019).

Inverse correlations of FHB Type I and II resistances with plant height (PLHT) have been reported (Paillard et al., 2004; Schmolke et al., 2005). Further, increased FHB symptoms have been observed in genotypes carrying the reduced plant height genes, *Rht1* (*Rht-B1*) on chromosome 4B (Gale et al., 1975), *Rht2* (*Rht-D1*) on chromosome 4D (Gale and Marshall, 1976), or *Rht8* on chromosome 2D (Hilton et al., 1999; Steiner et al., 2004; Miedaner and Voss, 2008; Srinivasachary et al., 2008; Srinivasachary et al., 2009; Mao et al., 2010). Regarding days to anthesis (DTA), McCartney et al. (2007) found that FHB resistance QTLs do not have major effects on anthesis date and suggested that improvements in FHB resistance could be achieved without adverse changes in DTA. Further, evaluation studies of FHB Type II resistance under field conditions have been undertaken using point inoculations, and back-pack and tractor-mounted sprays (Buerstmayr et al., 2002; Buerstmayr et al., 2003), mainly taking into consideration the environmental conditions around anthesis and rarely the effect of DTA per se. A recent study by Franco et al. (2021) used DTA as a covariate or source of variation in a prediction model for spring wheat FHB Type II resistance in several field environments and found DTA to explain 26% of the total phenotypic variation in FHB severity or Type II resistance.

Among approaches to identify FHB resistances, enhance breeding selection, and eventually limit losses, marker-assisted selection (MAS) is widely used (Bai and Shaner, 2004; Anderson, 2007; Miedaner and Korzun, 2012). Genome-wide association studies (GWASs) of FHB resistance (Buerstmayr et al., 2020), PLHT, and DTA can help detect trait-specific loci for the characterization of FHB in relation to PLHT and DTA (Sari et al., 2018; Sari et al., 2020). An understanding of the types of association (i.e., linkage or pleiotropy) between loci for FHB resistance and the said traits is essential in designing predictive markers for MAS (Sari et al., 2020). Quantitative trait nucleotides (QTNs) (Long and Langley, 1999) are polymorphic sites in genes corresponding to the QTLs and are responsible for variation in the trait phenotype (Mackay, 2001). When compared to linkage studies, GWAS has a greater power to detect associations between a QTN and the trait phenotype, even after correcting for multiple tests for association (Risch and Merikangas, 1996; Mackay, 2001). Multi-locus GWAS models (Wang et al., 2016a, Wang et al., 2016b) quantify the effects of multiple loci and are hence better suited for the analyses of quantitative traits when compared to single-locus models, which might often fail to detect small-effect loci influencing complex traits (Lan et al., 2020; Fernandes et al., 2022). The objectives of this study were to perform multi-locus GWAS analyses of a bread wheat association mapping panel (AMP) in order to a) detect QTNs that would help characterize FHB resistance, in relation to DTA and PLHT, and b) provide detail on physical locations of identified QTNs and their corresponding high-confidence candidate genes, as well as other proximal disease resistance, maturity, and PLHT genes.

## Materials and methods

### Plant material and multi-environment trials

An AMP comprising 192 predominantly Canadian spring wheat cultivars was assessed for FHB INC and SEV in multi-environment trials (METs) conducted between 2015 and 2017 at the three Agriculture and Agri-Food Canada (AAFC) FHB-inoculated nurseries near Morden (MDN) and Brandon (BDN) in Manitoba, and Ottawa (OWA) in Ontario, Canada. The panel comprised cultivar Sumai 3 with superior FHB resistance and its Canadian derivatives (AAC Brandon, AAC Elie, Cardale, and AC Carberry), derived from parents Alsen and ND744 (Zhu et al., 2019). However, both parents Alsen and ND744 are not part of the AMP. Further, the panel also comprised Brazilian cultivar Frontana, its derivative Neepawa, and Neepawa derivatives Stettler, Katepwa, and AC Barrie, in addition to 75 lines taken from registration trial collections of Canadian Western and Central Bread Wheat, Parkland, General Purpose, Hard White, and High Yielding Wheat (**Supplementary Material 1**). At all nursery sites, entries were replicated twice in a randomized complete block design (RCBD). A single dataset on INC, SEV, DTA, and PLHT was generated for each of the six environments (MDN 2015, MDN 2016, MDN 2017, BDN 2015, BDN 2016, and OWA 2017), with the exception of the MDN 2015 environment, which had two datasets. Hence, a total of seven MET datasets from six environments were utilized for the GWAS analyses. These included four datasets from MDN (MDN15-1, MDN15-2, MDN16, and MDN17), two from BDN (BDN15 and BDN16), and one from OWA (OWA17). The two MDN 2015 environment datasets (MDN15-1 and MDN15-2) on FHB INC and SEV were recorded a few days apart.

### FHB inoculum and disease assessment

To facilitate the development of FHB disease symptoms, artificial inoculation with *F. graminearum* isolates was carried out at the MET nursery sites, as described in Ruan et al. (2020). Briefly, 2–3 weeks prior to heading or when early lines were at the four- to five-leaf stage, corn spawn inoculum containing a mixture of four *F. graminearum* isolates [HSW-15-39 (3-ADON), HSW-15-87 (3-ADON), and HSW-15-27 (15-ADON), and HSW-15-57 (15-ADON)] from Dr. Henriquez’s FHB culture collection was applied at AAFC’s Morden and Brandon FHB nurseries; the inoculum mixture was spread (8 g/m row) twice, 1 week apart. Plots were irrigated thrice a week using Cadman Irrigation travellers with Briggs booms at Morden and with an overhead irrigator system at Brandon. At the Ottawa nursery, *F. graminearum* inoculum was prepared at a 1:1 ratio mixture of corn and barley kernels inoculated with three isolates [DAOMC178148 (15-ADON chemotype), DAOMC212678 (15-ADON chemotype), and DAOMC232369 (3-ADON chemotype)] obtained from the Canadian Collection of Fungal Cultures at the Ottawa Research and Development Centre. The three isolates collected locally were chosen for their high DON-producing capacity. Inoculation with 12 g of fresh inoculum was performed twice, with the first application occurring when the earliest lines started stem elongation, before flag leaf emergence (approx. Zadoks stage 31–36), and again 2 weeks later. Plots were irrigated daily applying approximately 1.5 cm of rain equivalent with wedge drive impact sprinklers. At approximately 21 days post-anthesis, the proportion of infected spikes per row (INC) and the average infected spikelets per head (SEV) were assessed visually and recorded as a percentage on a 0–100 scale (Stack and McMullen, 1985). The FHB disease index (IND) value for a given genotype was deduced from its INC and SEV ratings using the formula INC × SEV/100 and expressed as a percentage. The MDN 2015 environment had two datasets (MDN15-1 and MDN15-2), each with INC and SEV ratings recorded a few days apart. PLHT and DTA were recorded only for the MDN 2015, 2016, and 2017 environments.

### Statistical data analyses

Statistical analyses of FHB datasets were performed separately for each of the three trial locations at BDN, MDN, and OWA. With the use of the *lme4* package (Bates et al., 2015) in R version 4.2.1 (R Development Core Team, 2019), best linear unbiased prediction (BLUP) values for INC, SEV, IND, DTA, and PLHT belonging to a single location, across multiple years, were fitted with a mixed linear model (M1) *via* the equation *Y_ij_* = *µ* + *G_i_* + *E_j_* + *GE_ij_* + *e_ij_*, where *Y_ij_* is the phenotypic trait values of genotype *i* in environment *j*, *µ* is the population mean, *G_i_* is the effect of genotype *i*, *E_j_* is the effect of environment (location-year) *j*, *GE_ij_* is the G × E interaction between genotype *i* and environment *j*, and *e_ij_* is the residual effect associated with genotype *i* in environment *j*. The restricted maximum likelihood (REML) method was used to determine variance components (VCs). VCs were used to obtain broad sense heritability (*H*^2^) estimates for individual traits in a given environment, where replication is nested within the environment, using the formula *H*^2^ = *σ*^2^*G*/(*σ*^2^*G* + *σ*^2^*GY*/*y* + *σ*^2^*GL*/*l* + *σ*^2^*GYL*/*yl* + *σ*^2^*e*/*ply*), where *σ*^2^*G* is the genetic variance; *σ*^2^*GY* is the variance of the Genotype × Year interaction; *σ*^2^*GL* and *σ*^2^*GYL* are the interaction variances of the Genotype × Location, and Genotype × Year × Location, respectively; and *σ*^2^*e* is the error variance. Further, *y* is the number of years in which trials were conducted, *l* is the number of trial locations, and *p* is the total number of replications per location. Three-year (2015–2017) data on PLHT and DTA from MDN were averaged separately and deployed in a correlation analysis with FHB traits for BDN and OWA environments. An analysis of variance (ANOVA) for FHB traits across environments was performed using the *aov* function of the dplyr package in R to determine the effects of genotype (G), environment (E), and genotype-by-environment (G × E) variances on FHB INC, SEV, and IND.

### DNA extraction, genotyping, and data processing

Genomic DNA (gDNA) extraction from freeze-dried seedling leaves of the 192 cultivars was performed using a cetyltrimethylammonium bromide (CTAB)-based method. An automated AutoGen DNA isolation system (AutoGen, Holliston, MA, USA) and a Quant-iT™ PicoGreen dsDNA Assay Kit (Thermo Fisher Scientific Inc., Bartlesville, OK, USA) were deployed to obtain final gDNA sample concentrations of 50 ng/μl for genotyping. The AMP was genotyped with the wheat 90K single-nucleotide polymorphism (SNP) Infinium^®^ Beadchip (Wang et al., 2014). SNP call raw data were curated and imported into Genome Studio Software to filter SNP markers with greater than 20% missing data and minor allele frequency (MAF) of less than 5%. After filtering, an initial 9,084 SNPs were obtained, of which a total of 5,441 SNPs with corresponding International Wheat Genome Sequencing Consortium (IWGSC) RefSeq v1.0 physical map locations were used in this study.

### Population structure, LD, kinship, and principal components

To infer the population genetic structure of the AMP, Structure^®^ software (Pritchard et al., 2000) was deployed to determine the true number of clusters (*K*) or sub-populations within the AMP, using the Evanno method (Evanno et al., 2005). Run-parameters included length of burn-in iterations and Markov chain Monte Carlo (MCMC) simulations of 10,000 each, for K = 2–9 clusters, with 10 replications per value of *K*. Output from Structure was processed with the Structure Harvester^®^ (Earl and VonHoldt, 2012) program and visualized on its website (https://taylor0.biology.ucla.edu/structureHarvester/). Structure Harvester uses an algorithm to execute the Evanno method, with a minimum of three sequential values of *K* and three replicates, and produces three plots, one of which uses a Delta K statistic to determine the number of *K* groups. To visualize relatedness or similarity among genotypes, a dimensionless Constellation plot depicting the hierarchical clustering patterns of the AMP was generated in JMP^®^ Version 17 (SAS Institute Inc., Cary, NC, USA). In TASSEL (Trait Analysis by aSSociation, Evolution, and Linkage) 5 (Bradbury et al., 2007), a genotypic file in the HapMap format, with physical map locations for 5,441 SNP markers and their corresponding calls on 192 lines, was uploaded. The HapMap file was masked and subject to a linkage disequilibrium K-number neighbor imputation (LDKNNi) algorithm (Money et al., 2015) to replace missing calls and improve the overall accuracy of the data. Next, the genotypic, genotypic-masked, and genotypic-masked LDKNNi files were combined and evaluated for imputation accuracy.

LD decay, principal component analyses (PCAs), and relatedness or kinship analyses were estimated in TASSEL 5. An LD statistics output file from TASSEL 5 was imported to R to generate a scatter plot using the *ggplot2* function. The scatter plot helped visualize and determine LD decay on a whole-genome basis. To estimate kinship, the HapMap genotype data file was uploaded to TASSEL 5 and processed to output a.*csv* kinship (K-matrix) file. For PCA, the HapMap, phenotypic data file, and a population structure Q-matrix file (from the Structure run) were uploaded to TASSEL 5 to generate a.*csv* PCA output file. Both PCA and kinship.*csv* files were used as input for the GWAS analyses.

### GWAS analyses

To identify loci associated with FHB traits, DTA, and PLHT, GWAS was performed separately for all six individual environments. Both single-locus mixed linear model (MLM) and multi-locus random SNP effect-mixed linear model (mrMLM) were deployed to compare and validate marker–trait associations. Further, to assess the consistency or reproducibility of QTNs detected from a multi-locus GWAS of individual environments, a combined multi-locus GWAS analysis using entry trait means based on all six environments was performed. With the use of the GAPIT (Genome Association and Prediction Integrated Tool) 3 package (Lipka et al., 2012; Tang et al., 2016; Wang and Zhang, 2021) in R, datasets were fitted with a single-locus MLM. Next, datasets were analyzed with an mrMLM (Wang et al., 2016a; Wang et al., 2016b), also performed in R with the mrMLM v4.0.2 software, which integrates six methods for QTN detection (Zhang et al., 2020). These six methods include mrMLM (Wang et al., 2016), FASTmrMLM (Tamba and Zhang, 2018), FASTmrEMMA (Wen et al., 2018), pLARmEB (Zhang et al., 2017), pKWmEB (Ren et al., 2018), and ISIS EM-BLASSO (Tamba et al., 2017). For both models, kinship (K-matrix) and PCA or Q-matrix.*csv* files, along with HapMap and phenotypic data files, were used as input for the GWAS analyses. In the mrMLM method, QTNs with a limit of detection (LOD) score greater than 3 and appearing in two or more environments were considered statistically significant. However, in the single-locus MLM method, QTNs were considered statistically significant if they exceeded a more stringent threshold of −log_10_(*p*) = 5, which is based on a critical *p*-value (α = 0.05) subject to a Bonferroni correction of 0.05/*n*, where *n* is the number of SNP markers (He et al., 2019).

### QTN nomenclature, physical mapping, and candidate gene identification

Statistically significant QTNs detected across all environments by single-locus MLM and multi-locus mrMLM methods were numbered from 1 to 291. In most cases, the same SNP was associated with one or more traits per environment, or across environments. Hence, to refer to all QTNs associated with a given trait or traits, as a single entity, the chromosomal location followed by QTN numbers is given in the QTN name. For example, *QTN4B_144-226* is the collective QTN name, which represents two PLHT QTNs (*144_Tdurum_contig42229_113* and *226_Tdurum_contig42229_113*) detected on chromosome 4B.

The IWGSC reference genome RefSeq v1.0 was used for the physical positioning of QTNs and candidate genes detected from the GWAS analyses. The RefSeq v1.0 was preferred over RefSeq v2.0 and the current RefSeq v2.1 since it provided all QTN-associated candidate gene annotations. However, for better context, the current/new physical locations of updated candidate gene IDs (as per RefSeq v2.1) have also been provided in all MET dataset tables. For candidate gene detection, 90K SNP nucleotide sequences were subject to a nucleotide-BLAST (BLASTn) basic search with an expected threshold of 0.0001 (maximum E-value 10^−4^) on the online IWGSC RefSeq v2.1 database (https://urgi.versailles.inrae.fr/blast_iwgsc). High-confidence *Traes* gene IDs (as per RefSeq v1.0) were each searched in Ensemble Plants (http://plants.ensembl.org/index.html) and UniProt (https://www.uniprot.org/) databases to obtain their corresponding gene functional descriptions.

## Results

### Variation in FHB, DTA, and PLHT trait phenotypes

Among the seven MET datasets from the Brandon, Morden, and Ottawa nurseries (**Supplementary Material 1**), OWA17 had the highest mean FHB INC rating of 85.1%, followed by overall mean ratings of 78.9% at BDN and 68.8% at MDN (**Table 1**). For FHB SEV, the highest overall mean rating of 56.1% was observed at BDN, followed by 42.4% at MDN and a mean SEV rating of 41.2% for the OWA 2017 environment. Overall mean ratings for FHB IND were the highest (47.8%) at BDN, followed by 30.3% at MDN and 36% at OWA. Broad-sense heritability for FHB measurements ranged from 55% to 82% for INC, 56% to 83% for SEV, and 58% to 83% for IND. For the MDN 2015 (MDN15-1 dataset), MDN 2016, and MDN 2017 environments, the overall means for DTA was 72 days (ranging from 47 to 72 days) and 89.7 cm for PLHT (ranging from 44 to 121 cm). Across environments, broad-sense heritability (*H*^2^) observed for PLHT was 92% and 87% for DTA (**Table 1**).

**Table 1:** Range, mean, standard deviation (SD), coefficient of variation (CV), and broad-sense heritability (*H*^2^) values for FHB incidence (INC), severity (SEV), index (IND), days to anthesis (DTA), and plant height (PLHT) from trial datasets of the AAFC nurseries at Ottawa (OWA), Brandon (BDN), and Morden (MDN) between 2015 and 2017.

Location	FHB trait	Year	Range		Mean	SD	CV	*H*^2^
			Min.	Max.				
Brandon	INC	2015	5	100	85.1	18.9	0.22	0.55
(BDN)		2016	0	100	72.8	25.2	0.35	
		Overall	0	100	78.9			
	SEV	2015	10	100	65.1	23.8	0.36	0.70
		2016	0	100	47.1	28.1	0.59	
		Overall	0	100	56.1			
	IND	2015	0.8	100	58.6	27.5	0.47	0.67
		2016	0	100	36.9	26.5	0.72	
		Overall	0	100	47.8			
Morden	INC	2015-1	0	90	53.3	21.2	0.39	0.82
(MDN)		2015-2	5	100	60.5	19	0.31	
		2016	0	100	83.7	17.2	0.21	
		2017	10	100	77.8	20.5	0.26	
		Overall	0	100	68.8			
	SEV	2015-1	0	90	42.1	19.1	0.45	0.83
		2015-2	5	95	45.7	18.8	0.41	
		2016	0	90	44	17.6	0.4	
		2017	10	90	37.9	16	0.42	
		Overall	0	95	42.4			
	IND	2015-1	0	81	24.7	16.6	0.67	0.83
		2015-2	0.5	90	30	18.4	0.61	
		2016	0	85	37.8	17.6	0.46	
		2017	2	78	28.5	15.4	0.54	
		Overall	0	90	30.3			
	DTA	2015-1	47	61	49.8	3	0.06	0.87
	(days)	2016	49	67	58.4	3.3	0.06	
		2017	57	72	63.3	2.7	0.04	
		Overall	47	72	57.2			
	PLHT	2015-1	61	113	86.5	8.8	0.1	0.92
	(cm)	2016	69	112	92.4	7.9	0.09	
		2017	44	121	90.2	9.2	0.1	
		Overall	44	121	89.7			
Ottawa	INC	2017	10	95	85.1	12.2	0.144	0.61
(OWA)	SEV	2017	5	85	41.2	15.5	0.38	0.56
	IND	2017	0.5	81	36	15.5	0.43	0.58

DTA and PLHT data were collected only from MDN.

**Figure 1:**
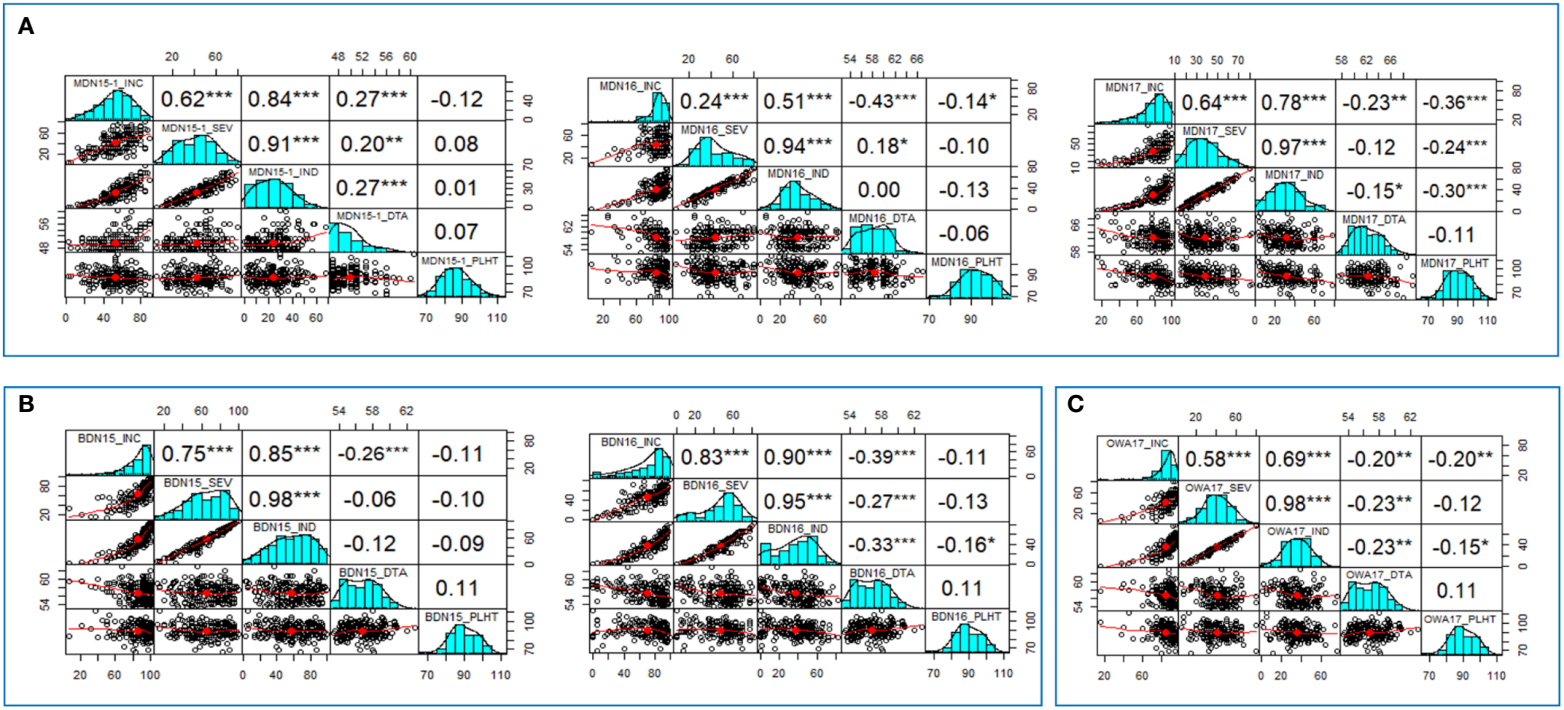
Correlation values and graphs generated from **(A)**. Morden (MDN15-1, MDN16, MDN17); **(B)**. Brandon (BDN15, BDN16) and **(C)**. Ottawa (OWA17) datasets, depicting relationships between FHB incidence (INC), Severity (SEV), Index (IND), days to anthesis (DTA) and plant height (PLHT) traits recorded on 192 bread wheat genotypes of a GWAS panel.

In the three MDN environments (MDN 2015, 2016, and 2017), positive and highly significant correlations ranging from 0.24 to 0.97 were observed among FHB INC, SEV, and IND traits. Overall weak, inverse correlations were observed between INC and PLHT (−0.14 to −0.36), SEV and PLHT (−0.24), and IND and PLHT (−0.30). DTA had overall weak inverse correlations with INC and with IND (−0.23 to −0.43), as well as weak positive correlations (0.18 to 0.2) with SEV (**Figure 1**). For FHB traits at the two BDN environments (BDN 2015 and 2016), highly significant and strong correlations ranging between 0.75 and 0.98 were observed between IND, SEV, and IND. Overall, weak, inverse correlations were observed between IND and PLHT (−0.16), INC and DTA (−0.26 to −0.39), SEV and DTA (−0.27), and IND and DTA (−0.33). In the OWA 2017 environment, highly significant and moderate-to-strong correlations of 0.58–0.98 were observed between INC, SEV, and IND traits. Finally, weak inverse correlations were observed between INC and PLHT (−0.20), IND and PLHT (−0.15), INC and DTA (−0.20), SEV and DTA (−0.23), and IND and DTA (−0.23; **Figure 1**). An ANOVA performed across environments revealed significant effects of genotype (G), environment (E), and G × E interactions on FHB INC, SEV, and IND (**Table 2**).

**Table 2:** An analysis of variance (ANOVA) for FHB incidence (INC), severity (SEV), and index (IND) traits across six environments of AAFC nurseries located at Morden (MDN) and Brandon (BDN) in Manitoba (MB) and Ottawa (OWA) in Ontario (ON) Canada, between 2015 and 2017.

Source of Variance	DF	INC	SEV	IND
Genotype (G)	191	1468.2**	1,731.5**	1,850.2**
Environment (E)	5	57,445.6**	33,971.1**	50,387.8**
G × E	954	3,34.9**	334.4**	349.2**
Residuals	1151	240	275.9	241.6

* Significant at p < 0.05.

** Highly significant at p < 0.01.

### SNP distribution, population structure, and LD decay

Imputation of missing calls in the HapMap genotypic data file with 5,441 SNP markers *via* the LDKNNi algorithm in TASSEL produced an imputation accuracy of approximately 93% (0.069 error rate). The largest number of SNP markers (2422) was distributed across the B genome, followed by the A genome with 1,978 and the D genome with 1,041. On a per-chromosome basis, the most number of SNP markers (551) was mapped to 2B, while 4D had the least number (43) of SNPs. The SNP marker distribution across 21 bread wheat chromosomes is given in an SNP density plot depicting the number of SNP markers within a 1-Mb window (**Supplementary Figure 1**).

**Figure 2:**
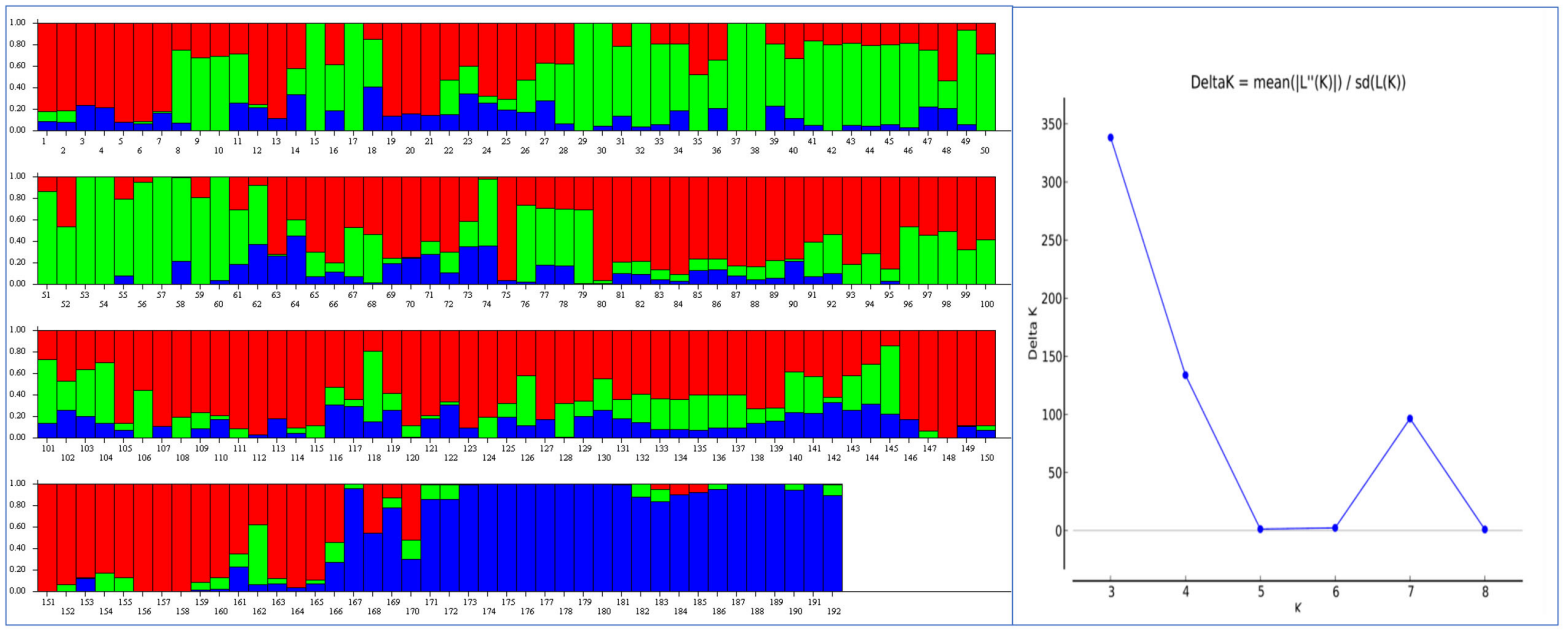
A population genetic structure analysis performed with the Structure® software detected three clusters (red, blue and green) or sub-populations (K=3) in an association mapping panel (AMP) comprising 192 predominantly Canadian spring wheat cultivars (*Left*), and an output graph visualized in Structure Harvester depicting the highest Delta K values obtained for K=3 (*Right*).

**Figure 3:**
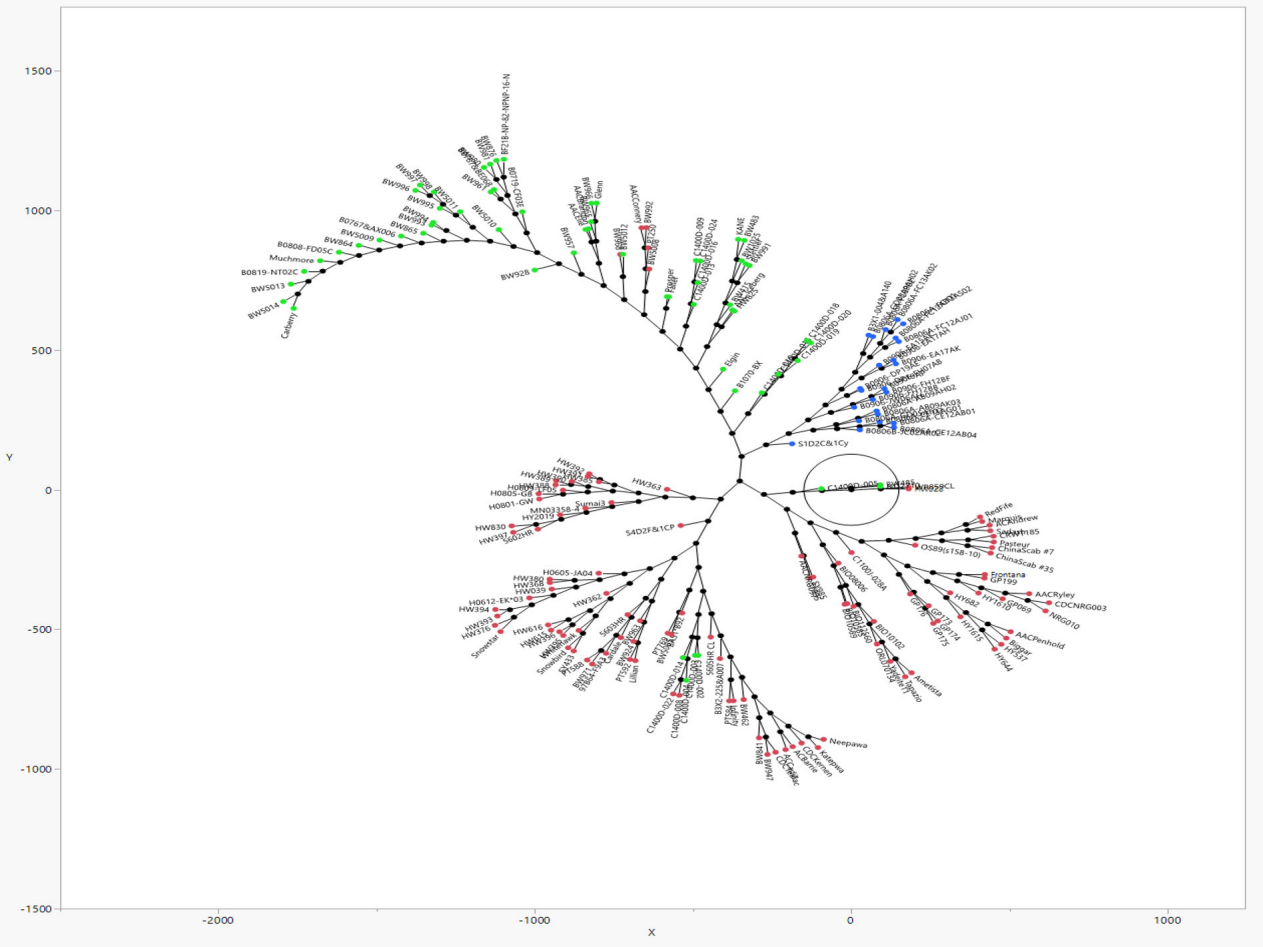
A dimensionless Constellation plot generated with JMP® Version 17 software, from hierarchical clustering of 192 predominantly Canadian spring wheats of an association mapping panel (AMP) genotyped with 5441 SNP markers. Three distinct clusters (red, blue, green) which correspond to the three sub-populations detected by Structure software (refer **Figure 2**), represent elite FHB tolerant cultivars of Asian and North American pedigree, in addition to Brazilian cultivar Frontana, its derivative Neepawa, and Neepawa derivatives Stettler, Katepwa, AC Barrie, besides 75 lines taken from Canadian Western Bread Wheat, Central Bread Wheat, Parkland Wheat, General Purpose Wheat, Hard White Wheat and High Yielding Wheat Registration trial collections.

A population genetic structure analysis with the Structure software revealed three clusters or sub-populations represented by red, blue, and green rectangular bars, among the 192 genotypes of the AMP, based on the highest Delta K value corresponding to K = 3 (**Figure 2**). This was validated by a dimensionless Constellation plot with three distinct clusters (red, blue, and green) generated in JMP^®^ Version 17 (SAS Institute Inc., Cary, NC, USA) (**Figure 3**). LD is described here as the r^2^ of marker pairs versus the genetic distance in base pairs (bp) across the genome. In the association panel, LD decay below a threshold of 0.2 r^2^ was observed to occur at a physical distance of ~7.08 Mb (**Supplementary Figure 2**).

### GWAS analyses

#### Single-locus GWAS (MLM)

**Figure 4:**
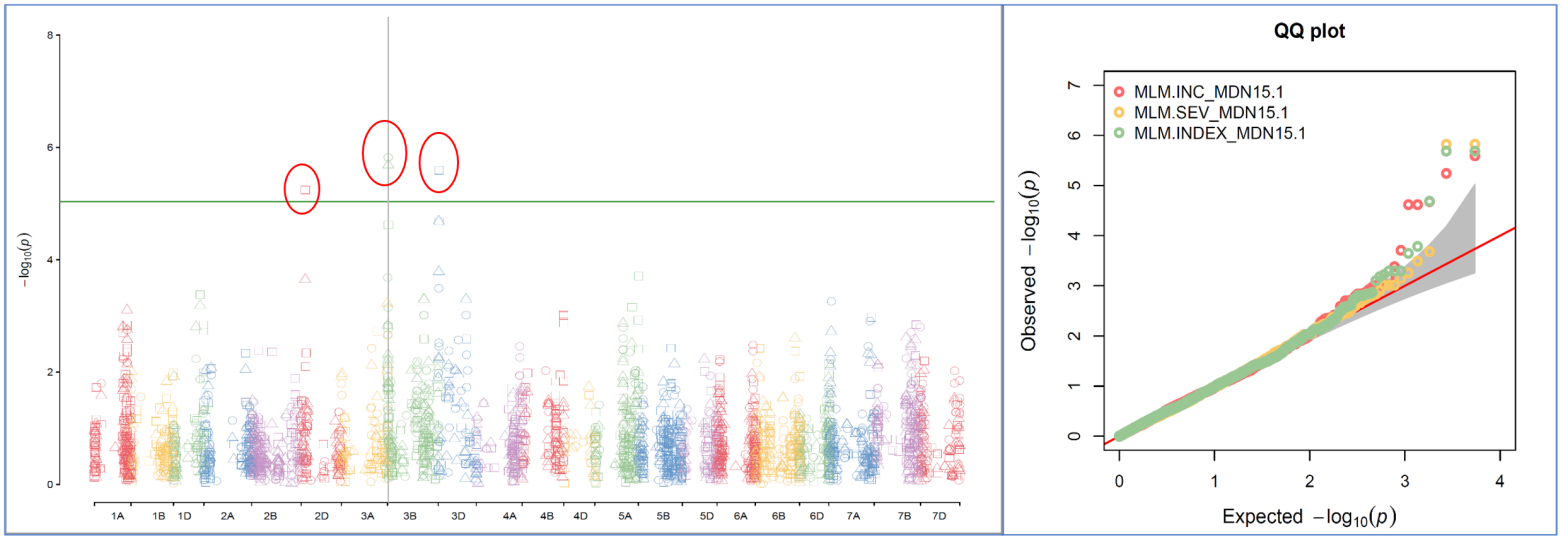
A multiple symphysic Manhattan plot generated from a MDN15.1 dataset (Morden 2015 environment) by the single-locus Mixed Linear Model (MLM) GWAS method depicts four significant Quantitative Trait Nucleotide (QTN; within three red ellipses) for FHB Incidence (INC) on chromosomes 2D and 3D, and for FHB Severity (SEV) and FHB Index (INDEX) on chromosome 3B (Left); and their corresponding probability distributions given in a symphysic Quantile-Quantile (Q-Q) plot (Right). The green horizontal line represents the significance threshold cutoff (-log_10_ (p) = 5). Note: The SEV and IND QTN on chromosome 3B are located within the Fhb1 region, and are represented by SNP marker *CAP7_c1576_371* which co-locates with gene *TraesCS3B02G024900* encoding a DEAD/DEAH box RNA helicase.

Single-locus MLM method revealed four statistically significant QTNs (−log_10_(*p*) > 5) for FHB INC on chromosomes 2D and 3D, and for SEV and IND on chromosome 3B, only from two MDN 2015 environment datasets (MDN15-1 and MDN15-2). The SEV and IND QTNs detected on chromosomes 3B in both datasets are identical and represented by SNP marker *CAP7_c1576_371*, which co-locates with *TraesCS3B02G024900*, which encodes a DEAD/DEAH box RNA helicase domain or *TraesCS3B03G0056500* (3B:15817353..15827081) in RefSeq v2.1 (**Figure 4**). When all site years were considered, a total of 242 statistically significantly (*p* < 0.05) SNP markers explained 17%–26% of the phenotypic variance for INC, 264 SNPs explained 19%–35% of the variance for SEV, and 270 statistically significant SNP markers explained 23%–36% phenotypic variance for IND (**Supplementary Material 2**).

#### Multi-locus GWAS (mrMLM)

In seven MET datasets from six environments, mrMLM v4.0.2 software detected a total of 291 statistically significant QTNs (LOD score >3) for INC, SEV, DTA, and PLHT, across nearly all chromosomes. In the environment-wise distribution of the 291 QTNs, 50 were for INC, 72 for SEV, 90 for IND, 41 for DTA, and 38 for PLHT, as given in **Table 3**. Physical locations, high-confidence candidate genes (as per RefSeq 1.0 and 2.1), LOD scores, and *p*-values of all QTNs are given in **Supplementary Material 3**. Of these 291, 17 QTNs for FHB traits (INC, SEV, and IND), DTA, and PLHT were detected in two or more environments at MDN and BDN between 2015 and 2017. Twelve of these 17 QTNs were pleiotropic for a combination of traits, i.e., INC, SEV, IND, DTA, or PLHT (**Table 4**). Further, combined multi-locus GWAS analyses using entry trait means based on all six environments detected a total of 79 statistically significant QTNs for FHB traits, DTA, and PLHT (**Supplementary Material 4**), 15 of which were identical to 15 of the 17 statistically significant QTNs detected at two or more environments. Of the 15 combined analysis QTNs, three were for INC (on chromosomes 2D, 5A, and 7b), three for SEV (on chromosomes 3B, 5A, and 7B), five for IND (on chromosomes 3B, 5A, 6B, 7A, and 7B), one for DTA (on chromosome 1B), and three for PLHT (on chromosomes 4B, 6B, and 7B). Further, three of these 15 QTNs were pleiotropic for FHB INC, SEV, and IND on chromosome 5A; SEV and IND on chromosome 3B; and FHB INC, SEV, IND, and PLHT on chromosome 7B (**Table 4**).

**Table 3:** Environment-wise distribution of 291 quantitative trait nucleotides (QTNs) for FHB incidence (INC), severity (SEV), index (IND), days to anthesis (DTA), and plant height (PLHT) detected by the multi-locus random single-nucleotide polymorphism (SNP)–effect mixed linear model (mrMLM) genome-wide association study (GWAS) method in an association mapping panel (AMP) of 192 predominantly Canadian bread wheat genotypes using trait-data from six environments (seven datasets): Morden 2015 (MDN15-1 and MDN15-2), Brandon 2015 (BDN15), Brandon 2016 (BDN16), Morden 2016 (MDN16), and Morden 2017 (MDN17) in Manitoba and Ottawa 2017 (OWA17) in Ontario Canada.

Sl.	Environment	Dataset	Trait	No. of QTN	QTN located on chromosomes
1	Morden 2015	MDN15-1^a^	INC	13	1A, 1D, 2B, 2D, 3A, 3B, 3D, 5D, 7A, 7B
		MDN15-2^b^		10	1D, 2D, 3B, 3D, 5A, 6A, 7A, 7B
		MDN15-1	SEV	14	1A, 1D, 3B, 5A, 6A, 6B, 7A, 7B
		MDN15-2		11	1B, 1D, 2B, 3B, 5A, 6B, 6D, 7B
		MDN15-1	IND	18	1B, 1D, 2B, 2D, 3B, 3D, 5A, 5D, 6B, 7A, 7B
		MDN15-2		14	3B, 3D, 4A, 5A, 5B, 6A, 6B, 6D, 7B
		MDN15-1	PLHT ^c^	12	1B, 2B, 2D, **4B**, 5A, 5B, 6B, 6D, 7B
		MDN15-1	DTA	16	1A, 1B, 1D, 2A, 2B, 3B, 4A, 4B, 6A, 6B, 6D, 7A
2	Brandon 2015	BDN15	INC	–	–
			SEV	8	1A, 2A, 3A, 4A, 5B, 6B, 7B
			IND	2	1D, 6B
			PLHT	–	–
			DTA	–	–
3	Brandon 2016	BDN16	INC	17	1A, 1D, 2A, 2D, 3B, 3D, 5A, 5B, 6B, 7A, 7B
			SEV	9	3B, 4B, 6A, 6B, 7A, 7B
			IND	15	1B, 1D, 2D, 3D, 4B, 6A, 6B, 6D, 7A, 7B
			PLHT	–	–
			DTA	–	–
4	Morden 2016	MDN16	INC	2	1A, 5A
			SEV	4	2B, 2D, 5B, 6A
			IND	8	1A, 1B, 2B, 5A, 6A, 6D
			PLHT	13	1D, 4A, **4B**, 5A, 5B, 6A, 6B, 7B
			DTA	9	1A, 1B, 1D, 2D, 5B, 6B, 7A
5	Morden 2017	MDN17	INC	3	1B, 2A, 6A
			SEV	21	1A, 1B, 2A, 2B, 2D, 3A, 3B, 4A, 5A, 5B, 6B, 6D, 7A, 7B, 7D
			IND	25	1A, 1B, 2B, 2D, 3B, 4A, 4D, 6A, 6B, 6D, 7A, 7B, 7D
			PLHT	13	1B, 1D, 2A, **4B**, 6A, 6B, 7B, 7D
			DTA	16	1A, 1B, 2B, 2D, 3B, 4A, 5A, 5B, 5D, 6B, 7A
6	Ottawa 2017	OWA17	INC	5	1A, 3B, 6A, 6B
			SEV	5	3B, 5B, 6A, 6B
			IND	8	1A, 3B, 5B, 6A, 6B
			PLHT	–	–
			DTA	–	–

a First dataset on FHB INC and SEV recorded at the Morden 2015 environment.

b Second dataset on FHB INC and SEV recorded a few days later in the same Morden 2015 environment.

c A PLHT QTN, QTN4B_65-145-227 on chromosome 4B (bold typeface, underlined), detected in all three years (2015, 2016, and 2017) at Morden.

**Table 4:** Seventeen QTN for FHB incidence (INC), severity (SEV), index (IND), days to anthesis (DTA), and plant height (PLHT), their corresponding high-confidence candidate genes, physical locations (IWGSC RefSeq 1.0 and 2.1), limit of detection (LOD) scores, and explained percent phenotypic variances (R^2^) detected by single-locus mixed linear model (MLM) and multi-locus random single-nucleotide polymorphism (SNP)–effect mixed linear model (mrMLM) genome-wide association study (GWAS) methods with 2015-2017 multi-environment (MET) datasets from the FHB nurseries at Morden (MDN) and Brandon (BDN) in Manitoba (MB) Canada.

Sl	QTN name	Envron.	Trait	QTN SNP	Ch	Physical loc (bp)	RefSeq 1.0 gene ID	Description	RefSeq 2.1 gene new ID	RefSeq 2.1 gene	QTN effect	LOD score	−log10(*p*)	R^2^ (%)	MAF	Genotype
										Physical loc (bp)						
	Pleiotropic QTN															
1	***QTN2D_7-31-162-184-280***	MDN15-1^a^	INC	7_wsnp_Ex_c6400_11123059	2D	62957090	TraesCS2D02G113600	Ribosome biogenesis protein NSA2 homolog	TraesCS2D03G0238100	65431554.65434070	−9.4 to 24.6	4.2–6.6	5–7.4	12.1–22.4	0.23	AG, GG
			IND	31_wsnp_Ex_c6400_11123059							−15.1	4.23	5.0	7.20	0.23	AG
		MDN17	SEV	162_wsnp_Ex_c6400_11123059							3.2	3.3–4.2	4.01–4.9	8.6–13.1	0.22	GG
			IND	184_wsnp_Ex_c6400_11123059							−6.2	3.11	3.8	7.7	0.23	AG
		BDN16	IND	280_wsnp_Ex_c6400_11123059							−6.7	5.1	5.9	11.9	0.22	GG
	Combined analyses QTN^c^	–	INC	C3_wsnp_Ex_c6400_11123059							−5.0 to −5.4	3.3–6.5	4.0–7.3	6.9–15.3	0.22	AG, GG
2	***QTN3B_9-16-32-77-89-96-164-185***	MDN15-1	INC	9_CAP7_c1576_371	3B	10708086	TraesCS3B02G024900	DEAD/DEAH box RNA helicase	TraesCS3B03G0056500	15817353.15827081	11-13.2	4.8–5.3	5.6–6.1	18.3–22.4	0.21	GG
			SEV	16_CAP7_c1576_371							14.6-15.4	8.7–11.4	5.8–12.7	29.9–49.1	0.21	GG
			IND	32_CAP7_c1576_371							11.2-13.2	7.6–11.9	5.7–12.9	29.3–34.1	0.21	GG
		MDN15-2^b^	INC	77_CAP7_c1576_371							6.2-7.7	3.3–4.6	4.01–5.4	10.6–10.9	0.21	GG
			SEV	89_CAP7_c1576_371							9.4-15.01	4.2–11	4.9–11.9	17.1–45.2	0.21	GG
			IND	96_CAP7_c1576_371							11.7	7.4–8	6–8.9	25.8–32.7	0.21	GG
		MDN17	SEV	164_CAP7_c1576_371							6.7-6.7	3.5–4.6	4.3–5.4	10.6–13	0.21	GG
			IND	185_CAP7_c1576_371							5.9	4.7	5.5	4.7	0.213	GG
	Combined analyses QTN	–	SEV	C23_CAP7_c1576_371							5.5-9.0	3.3–9.9	4.0–10.9	11.1–20.3	0.21	GG
	Combined analyses QTN	–	IND	C38_CAP7_c1576_371							7.6-8.1	5.9–6.9	6.7–7.7	21.5–21.6	0.21	GG
3	***QTN5A_80-102-130-260***	MDN15-2	INC	80_BS00000006_51	5A	706240306	TraesCS5A02G554200	Beta-amylase[ … ]	TraesCS5A03G1295800	710066497.710070348	5.1	4.4	5.2	3.3	0.2	AA
			IND	102_BS00000006_51							4.9	5.9	6.7	3.4	0.2	AA
		MDN16	IND	130_BS00000006_51							5.4	4.7	5.5	7.7	0.2	AA
		BDN16	INC	260_BS00000006_51							5.8	3.8	4.5	1.9	0.2	AA
	Combined analyses QTN	–	INC	C11_BS00000006_51							3.1-3.6	3.5–5.1	4.2–5.9	3.5–4.8	0.21	AA
	Combined analyses QTN	–	SEV	C26_BS00000006_51							4.4-9.8	5.3–8.1	6.1–9.0	5.7–10.5	0.21	AA
	Combined analyses QTN	–	IND	C39_BS00000006_51							4.0-10.2	4.2–6.2	4.9–7.0	3.5–10.1	0.21	AA
4	***QTN6B_92-106-272***	MDN15-2	SEV	92_BS00080544_51	6B	234560063	TraesCS6B02G197300	*S*-Adenosylmethionine synthase	TraesCS6B03G0506800	240608170.240610672	−4.8	3.7	4.4	6.3	0.2	GG
			IND	106_BS00080544_51							−7.0	6.3	7.1	10.9	0.2	GG
		BDN16	SEV	272_BS00080544_51							−5.1	3.5	4.2	3.3	0.2	GG
	Combined analyses QTN	–	IND	C40_BS00080544_51							−5.1 to −5.6	5.4–6.0	6.2–6.9	7.0–13.5	0.17	GG
5	***QTN7A_40-274-288-173-197***	MDN15-1	IND	40_BS00014126_51	7A	23328723	TraesCS7A02G050200	Multiple organellar RNA editing factor 2, chloroplastic	TraesCS7A03G0111800	24246669.24250241	6.6	3.5	4.2	6.0	0.4	CC
		BDN16	SEV	274_BS00014126_51							7.6	3.3	4.0	6.1	0.4	AC
			IND	288_BS00014126_51							8.3	4.7	5.4	6.2	0.4	AC
		MDN17	SEV	173_BS00014126_51							0.00	3.3	4.06	0.00	0.4	AC
			IND	197_BS00014126_51							10.2	7.1	8.01	16.7	0.4	AC
6	***QTN7A_200-266-273-287***	MDN17	IND	200_Ex_c24796_2499	7A	712056803	TraesCS7A02G533600	Actin–fragmin kinase catalytic domain-containing protein	TraesCS7A03G1297200	717563958.717569590	−5.39	3.4	4.17	5.74	0.3	AG
		BDN16	INC	266_Ex_c24796_2499							−7.9	3.7	4.4	7.3	0.3	AG
			SEV	273_Ex_c24796_2499							−11.3 to −12.8	8.3–9.5	9.2–10.5	18.2–24.7	0.3	AG
			IND	287_Ex_c24796_2499							−7.8	4.0	4.7	11.4	0.3	AG
	Combined analyses QTN	–	IND	C41_Ex_c24796_2499							−3.9 to −4.2	3.4–3.5	4.1–4.2	5.0–6.8	0.3	AG
7	***QTN7B_26-267***	MDN15-1	SEV	26_tplb0060b03_1008	7B	742464077	TraesCS7B02G486500	Photosystem II stability/assembly factor	TraesCS7B03G1307200	754941324.754943778	−3.8	3.3	4.0	1.3	0.2	GG
		BDN16	INC	267_tplb0060b03_1008							−6.6	4.0	4.7	1.6	0.2	GG
8	***QTN1A_46-252***	MDN15-1	DTA	46_RAC875_c16391_426	1A	508555158	TraesCS1A02G317000	Uncharacterized protein	TraesCS1A03G0787000	510051474.510054986	−1.0	4.8	5.6	13.3	0.3	GG
		BDN16	INC	252_RAC875_c16391_426							0.0	3.0	3.7	0.0	0.3	GG
9	***QTN1D_49-255***	MDN15-1	DTA	49_Kukri_c27717_316	1D	238060333	TraesCS1D02G166700	Pentatricopeptide repeat-containing protein	TraesCS1D03G0429200	240672206.240673769	1.2–1.3	3.4–3.6	4.1–4.3	6.1–13.2	0.5	AG, GG
		BDN16	INC	255_Kukri_c27717_316							−14.0	3.8	4.5	9.3	0.5	GG
10	***QTN6B_59-262***	MDN15-1	DTA	59_BobWhite_c47347_420	6B	558926756	TraesCS6B02G311900	ATP synthase mitochondrial F1 complex assembly factor 1	TraesCS6B03G0894300	567042315.567046184	−1.6	4.0	4.7	20.1	0.2	AA
		BDN16	INC	262_BobWhite_c47347_420							14.8	3.9	4.7	19.0	0.2	AA
11	***QTN6B_70-169-194***	MDN15-1	PLHT	70_BS00104265_51	6B	645659866	TraesCS6B02G372300	Protein coding	TraesCS6B03G1051600	653923276.653926026	4.04	4.9	5.69	11.7	0.24	AA
		MDN17	SEV	169_BS00104265_51							−5.2 to −6.9	5.4–5.5	6.2–6.3	5.6–13.9	0.24	AA
			IND	194_BS00104265_51							−3.81	3.38	4.1	1.98	0.24	AA
12	***QTN7B_13-45-83-94-108-154-176-201-276-291***	MDN15-1	INC	13_wsnp_RFL_Contig2136_1423367	7B	670278986	TraesCS7B02G402800	MMS19 nucleotide excision repair protein	TraesCS7B03G1084500	677195719.677206408	7.0	3.2	3.9	4.99	0.31	AA
			IND	45_wsnp_RFL_Contig2136_1423367							5.9	5.1	5.9	15.6	0.32	AA
		MDN15-2	INC	83_wsnp_RFL_Contig2136_1423367							8.2	3.7	4.41	13.2	0.31	AA
			SEV	94_wsnp_RFL_Contig2136_1423367							7.8–22.8	4.8–5.8	5.5–6.6	10.5–22.4	0.3	AG, AA
			IND	108_wsnp_RFL_Contig2136_1423367							7.3–22.8	3.0–7.1	3.7–8.0	11.3–30.6	0.3	AG, AA
		MDN16	PLHT	154_wsnp_RFL_Contig2136_1423367							−2.68	4.12	4.87	6.05	0.31	AG
		MDN17	SEV	176_wsnp_RFL_Contig2136_1423367							7.4–19.5	4.3–10.2	5.1–11.1	11.0–22.7	0.31	AG, AA
			IND	201_wsnp_RFL_Contig2136_1423367							9.5–20.9	5.3–7.9	6.1–8.8	10.7–28.2	0.31	AA
		BDN16	SEV	276_wsnp_RFL_Contig2136_1423367							9.9–22.7	4.5–4.54	5.3–5.31	7.6–13.8	0.31	AG
			IND	291_wsnp_RFL_Contig2136_1423367							8.8–24.6	4.6–5.3	5.3–6.1	8.4–21.6	1.31	AG
	Combined analyses QTN	–	INC	C18_wsnp_RFL_Contig2136_1423367							4.1–5.4	3.8–5.8	4.5–6.7	10.4–13.1	0.34	GG, AA
	Combined analyses QTN	–	SEV	C32_wsnp_RFL_Contig2136_1423367							4.6–6.7	3.5–6.1	4.2–6.9	7.1–19.2	0.31	AG, AA
	Combined analyses QTN	–	IND	C42_wsnp_RFL_Contig2136_1423367							5.9–7.0	4.9–5.3	5.7–6.1	15.2–25.7	0.31	AG, AA
	Combined analyses QTN	–	PLHT	C76_wsnp_RFL_Contig2136_1423367							−1.8	3.2	3.9	4.0	0.31	AG
	Non-pleiotropic QTN															
13	***QTN1B_48-206***	MDN15-1	DTA	48_Ku_c106533_550	1B	563675235	TraesCS1B02G336700	Disease resistance protein	TraesCS1B03G0922300	570333804.570337336	1.5–3.4	7.4–7.8	8.2–8.7	8.3–37.5	0.18	AA
		MDN17	DTA	206_Ku_c106533_550							1.4–3.5	6.5–7.6	7.4–8.5	12.4–46.3	0.18	AA
	Combined analyses QTN	–	DTA	C44_Ku_c106533_550							1.3–3.3	8.6–9.9	9.5–10.8	11.7–59.2	0.18	AA
14	***QTN7A_61-219***	MDN15-1	DTA	61_tplb0045p11_893	7A	675235632	TraesCS7A02G483900	Uncharacterized protein	TraesCS7A03G1171700	679827145.679827600	−0.54	3.1	3.81	3.41	0.32	AG
		MDN17	DTA	219_tplb0045p11_893							−1.03 to −1.2	4.4–4.5	5.2–5.3	11.5–14	0.32	GG, AG
15	***QTN4B_65-145-227***	MDN15-1	PLHT	65_IACX1632	4B	578043021	TraesCS4B02G292600	*N*-Acetyltransferase domain-containing protein	TraesCS4B03G0770000	577090863.577094213	−6.8 to −13.8	5.6–6.05	6.5–6.9	12.4–34.1	0.38	AG
		MDN16	PLHT	145_IACX1632							−3.7 to −10.7	3.3–4.7	4.04–5.5	8.5–24.4	0.38	AG
		MDN17	PLHT	227_IACX1632							−8.02	3.21	3.92	3.94	0.38	AG
	Combined analyses QTN	–	PLHT	C64_IACX1632							−3.9 to −11.5	3.3–5.0	4.1–5.8	10.0–15.6	0.38	GG, AG
16	***QTN4B_144-226***	MDN16	PLHT	144_Tdurum_contig42229_113	4B	38280619	TraesCS4B02G049800	Protein kinase domain-containing protein	TraesCS4B03G0107800	41016487.41020890	4.7–6.0	9.3–15.5	10.2–16.5	17.8–34	0.19	AA
		MDN17	PLHT	226_Tdurum_contig42229_113							6	7.6	8.5	27.4	0.19	AA
17	***QTN6B_151-229***	MDN16	PLHT	151_RAC875_c58425_331	6B	8781213	TraesCS6B02G014300	Uncharacterized protein	TraesCS6B03G0032500	10687959.10689500	−3 to −3.1	4.2–4.3	4.9–5.1	7.7–12.9	0.2	AG
		MDN17	PLHT	229_RAC875_c58425_331							−4.3 to −12.7	7.2–12.6	8.04–13.6	13.3–43.6	0.2	GG, AG
	Combined analyses QTN	–	PLHT	C70_RAC875_c58425_331							−3.1 to −4.9	3.8–8.4	4.6–9.3	7.7–39.7	0.2	GG, AG

a First dataset on FHB INC and SEV recorded for the Morden 2015 environment.

b Second dataset on FHB INC and SEV recorded a few days later for the same Morden 2015 environment.

c Fifteen of 79 QTNs detected from combined analyses (six environments) performed via the multi-locus mrMLM GWAS method.

### Pleiotropic QTN appearing in two or more environments

Of the 17 pleiotropic QTNs, 12 for INC, SEV, IND, DTA, or PLHT were detected in the MDN 2015, 2016, 2017, and BDN 2016 environments (**Table 4**). Seven of these 12 QTNs were pleiotropic for FHB INC, SEV, or IND; three were pleiotropic for INC and DTA; and two were pleiotropic for FHB traits (INC, SEV, or IND) and PLHT. Phenotypic variance (R^2^) for FHB traits of the seven pleiotropic QTNs ranged from 1.6% to 22.4% for INC, up to 49.1% for SEV, and from 3.4% to 34.1% for IND. The three pleiotropic QTNs for INC and DTA had R^2^ values ranging between 11.7% and 20.1% for DTA and up to 19% for INC. For the two QTNs pleiotropic for FHB traits and PLHT, R^2^ values ranged from 5.0% to 13.2% for INC, 5.6% to 22.7% for SEV, 2.0% to 30.6% for IND, and 6.1% to 11.7% for PLHT (**Table 4**).

### Pleiotropic QTN co-located with high-confidence candidate genes

**Figure 5:**
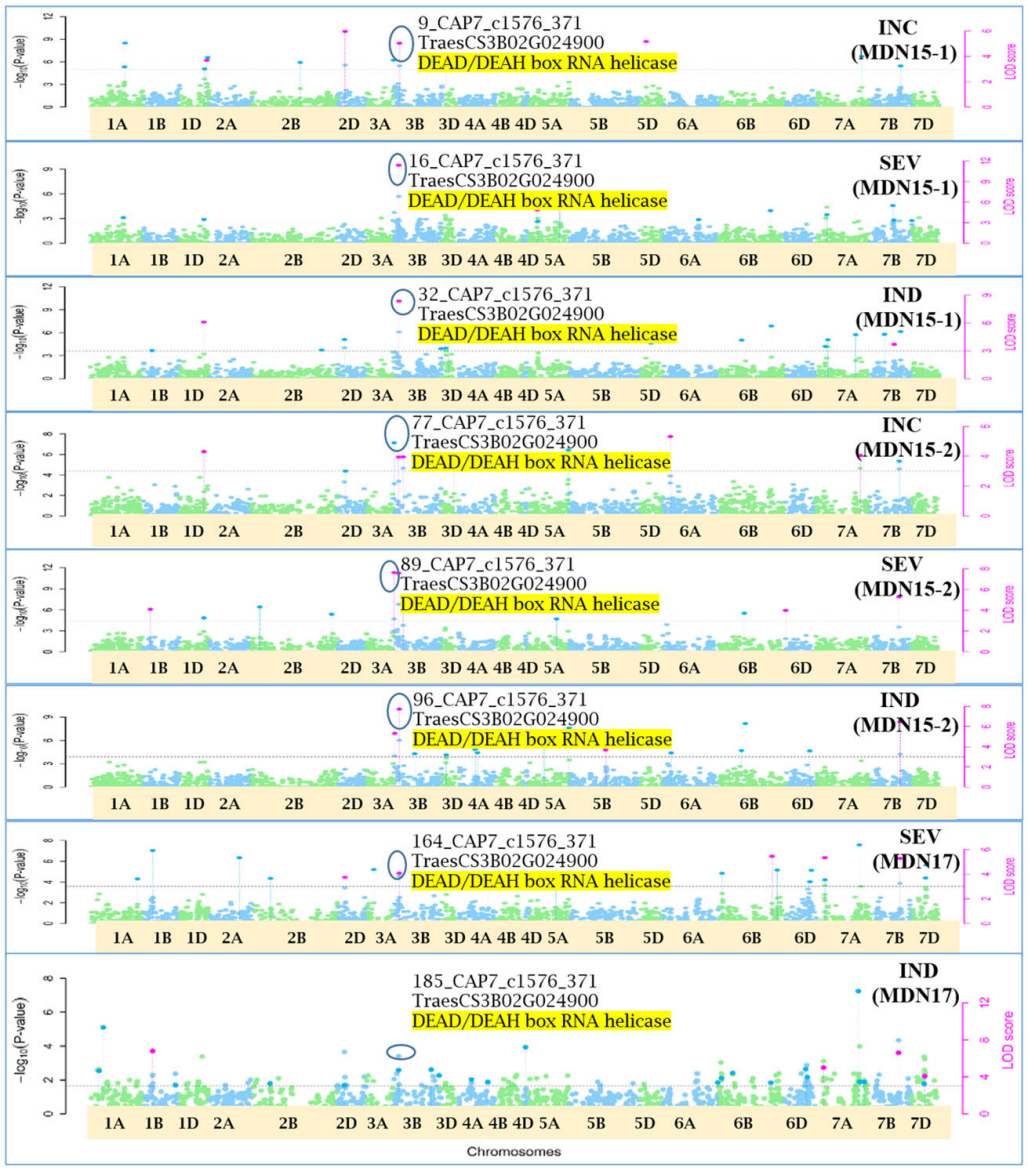
Manhattan plots depicting a pleiotropic Quantitative Trait Nucleotide (QTN), *QTN3B_9-16-32-77-89-96-164-185* for FHB Incidence (INC), Severity (SEV) and Index (IND) on chromosome 3B, represented by SNP marker *CAP7_c1576_371*, coinciding with a DEAD/DEAH box RNA helicase gene (*TraesCS3B02G024900*), detected from Morden 2015 (MDN15-1 & MDN15-2 datasets) (*top six plots*) and Morden 2017 (MDN17 datasets) environments (*bottom two plots*) by the multi-locus random SNP-effect Mixed Linear Model (MrMLM) method deployed on an association mapping panel of 192 predominantly Canadian bread wheats. The horizontal black dotted line denotes the significance threshold (LOD=3), while pink dots above the threshold line represent QTN detected by more than one of the six multi-locus methods.

**Figure 6:**
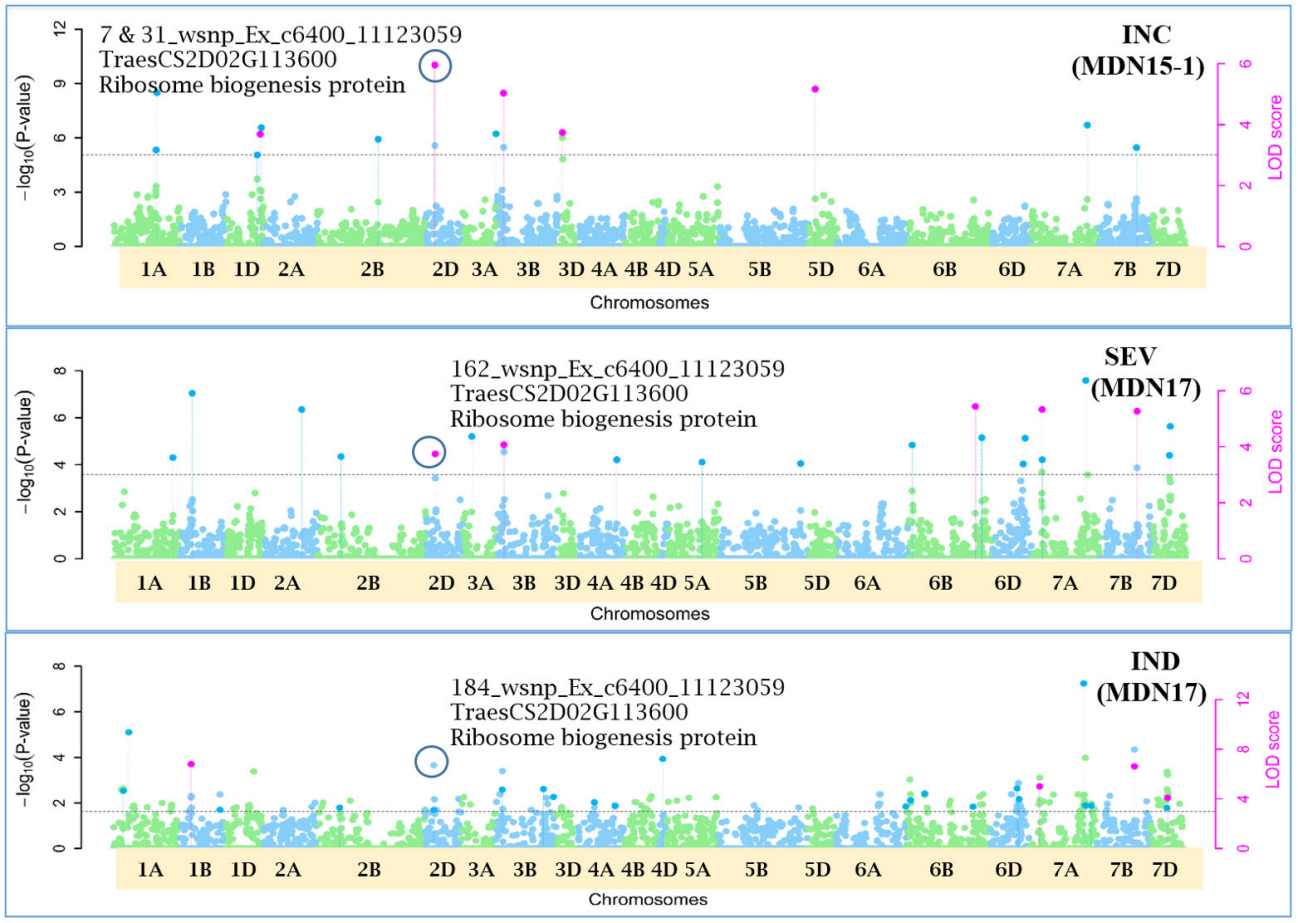
Manhattan plots depicting a pleiotropic Quantitative Trait Nucleotide (QTN), *QTN2D_7-31-162-184* for FHB Incidence (INC), Severity (SEV) and Index (IND) on chromosome 2D, represented by SNP marker *wsnp_Ex_c6400_11123059*, coinciding with a Ribosome biogenesis protein (*TraesCS2D02G113600*), detected from Morden 2015 (MDN15-1 dataset) (*top*) and Morden 2017 (MDN17 datasets) environments (*center and bottom*) by the multi-locus random SNP-effect Mixed Linear Model (MrMLM) method deployed on an association mapping panel of 192 predominantly Canadian bread wheats. The horizontal black dotted line denotes the significance threshold (LOD=3), while pink dots above the threshold line represent QTN detected by more than one of the six multi-locus methods.

**Figure 7:**
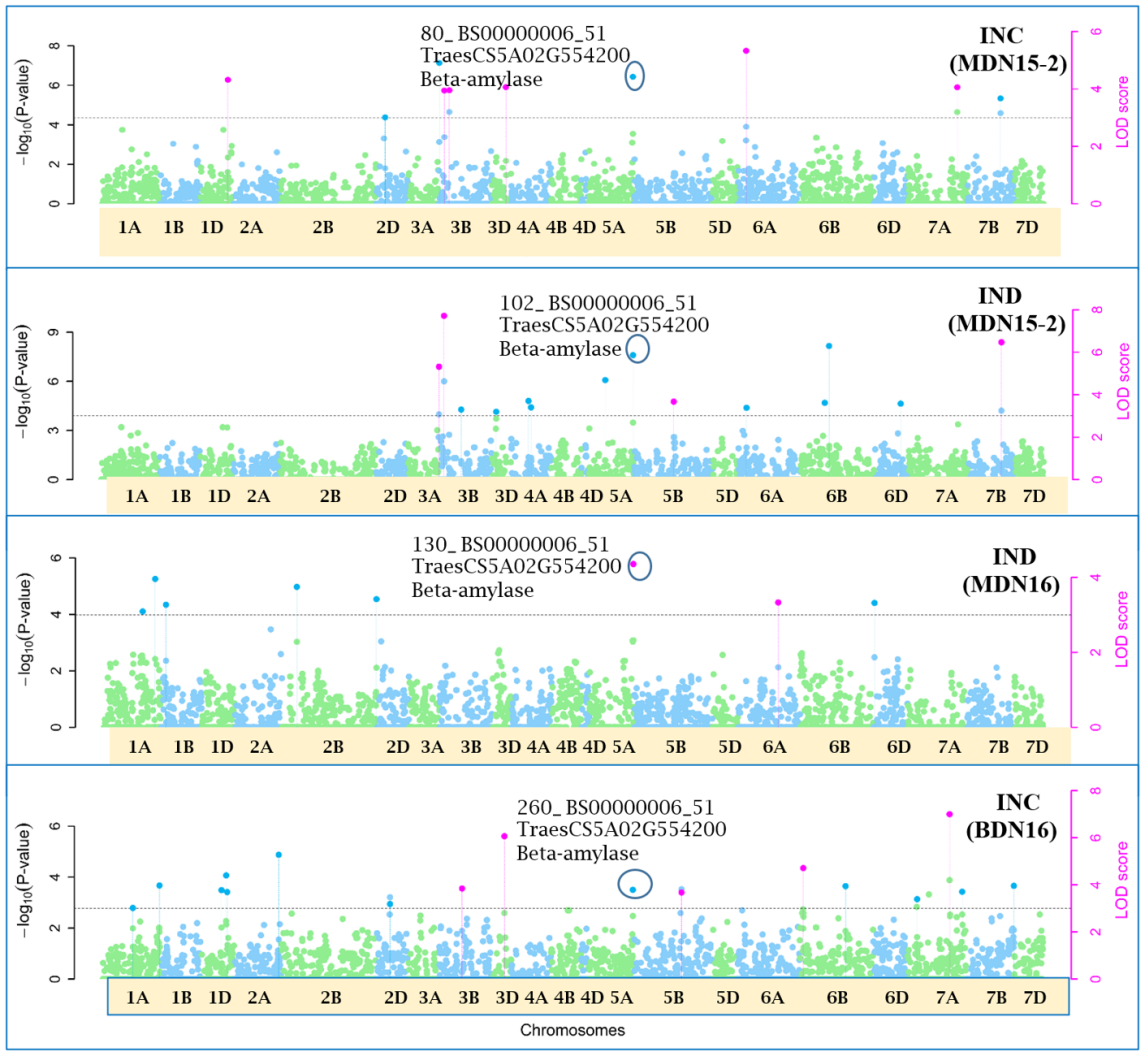
Manhattan plots depicting a pleiotropic Quantitative Trait Nucleotide (QTN) *QTN5A_80-102-130-260* for FHB Incidence (INC) and Index (IND) on chromosome 5A, detected in MDN 2015 (MDN15-2 dataset), MDN 2016 and BDN 2016 environments by the multi-locus random SNP-effect Mixed Linear Model (mrMLM) method deployed on an association mapping panel of 192 predominantly Canadian bread wheats. *QTN5A_80-102-130-260* is represented by SNP marker *BS00000006_51* which co-locates with an Beta-amylase protein (*TraesCS5A02G554200*). The horizontal black dotted line denotes the significance threshold (LOD=3), while pink dots above the threshold line represent QTN detected by more than one of the six multi-locus methods.

**Figure 8:**
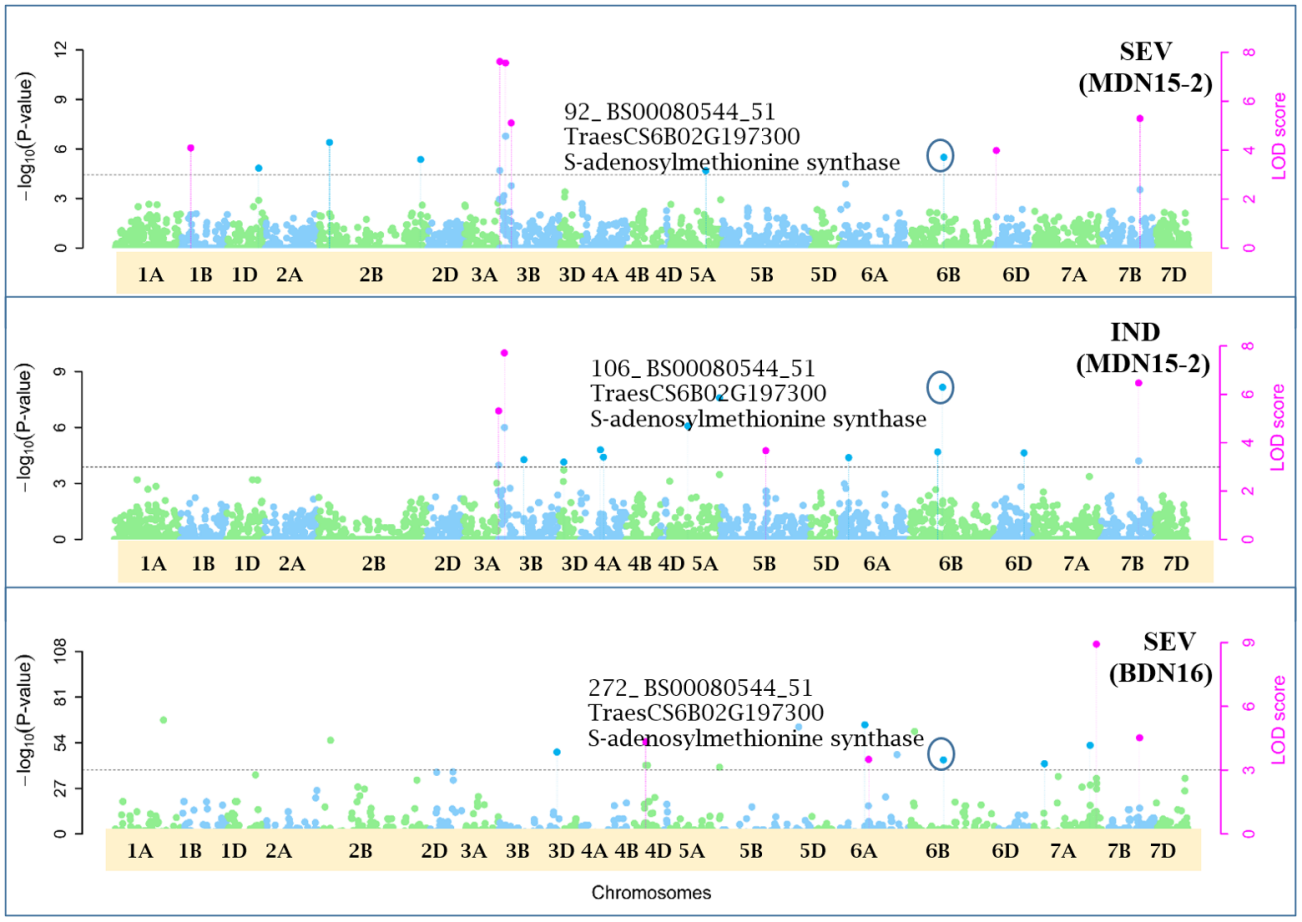
Manhattan plots depicting a pleiotropic Quantitative Trait Nucleotide (QTN) *QTN6B_92-106-272* for FHB Severity (SEV) and Index (IND) on chromosome 6B, detected in MDN 2015 (MDN15-2 dataset) and BDN 2016 environments by the multi-locus random SNP-effect Mixed Linear Model (mrMLM) method deployed on an association mapping panel of 192 predominantly Canadian bread wheats. *QTN6B_92-106-272* is represented by SNP marker *BS00080544_51* which co-locates with an S-adenosylmethionine synthase protein (*TraesCS6B02G197300*). The horizontal black dotted line denotes the significance threshold (LOD=3), while pink dots above the threshold line represent QTN detected by more than one of the six multi-locus methods.

**Figure 9:**
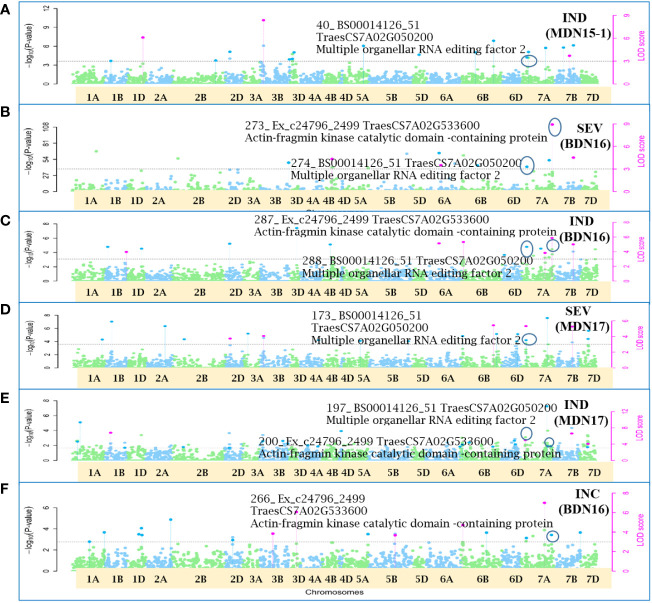
Manhattan plots depicting two pleiotropic Quantitative Trait Nucleotide (QTN) for FHB Incidence (INC), Severity (SEV) and Index (IND) on chromosome 7A, detected in MDN 2015 (MDN15-1 dataset), BDN 2016 and MDN 2017 environments by the multi-locus random SNP-effect Mixed Linear Model (mrMLM) method deployed on an association mapping panel of 192 predominantly Canadian bread wheats. Plots **(A–E)**: *QTN7A_40-274-288-173-197* for SEV and IND is represented by SNP marker *BS00014126_51* which co-locates with an Multiple organellar RNA editing factor 2 (*TraesCS7A02G050200*). Plots **(B, C, E, F)**: *QTN7A_200-266-273-287* for INC, SEV and IND is represented by SNP marker *Ex_c24796_2499* which co-locates with an Actin-fragmin kinase catalytic domain-containing protein (*TraesCS7A02G533600*). The horizontal black dotted line denotes the significance threshold (LOD=3), while pink dots above the threshold line represent QTN detected by more than one of the six multi-locus methods.

**Figure 10:**
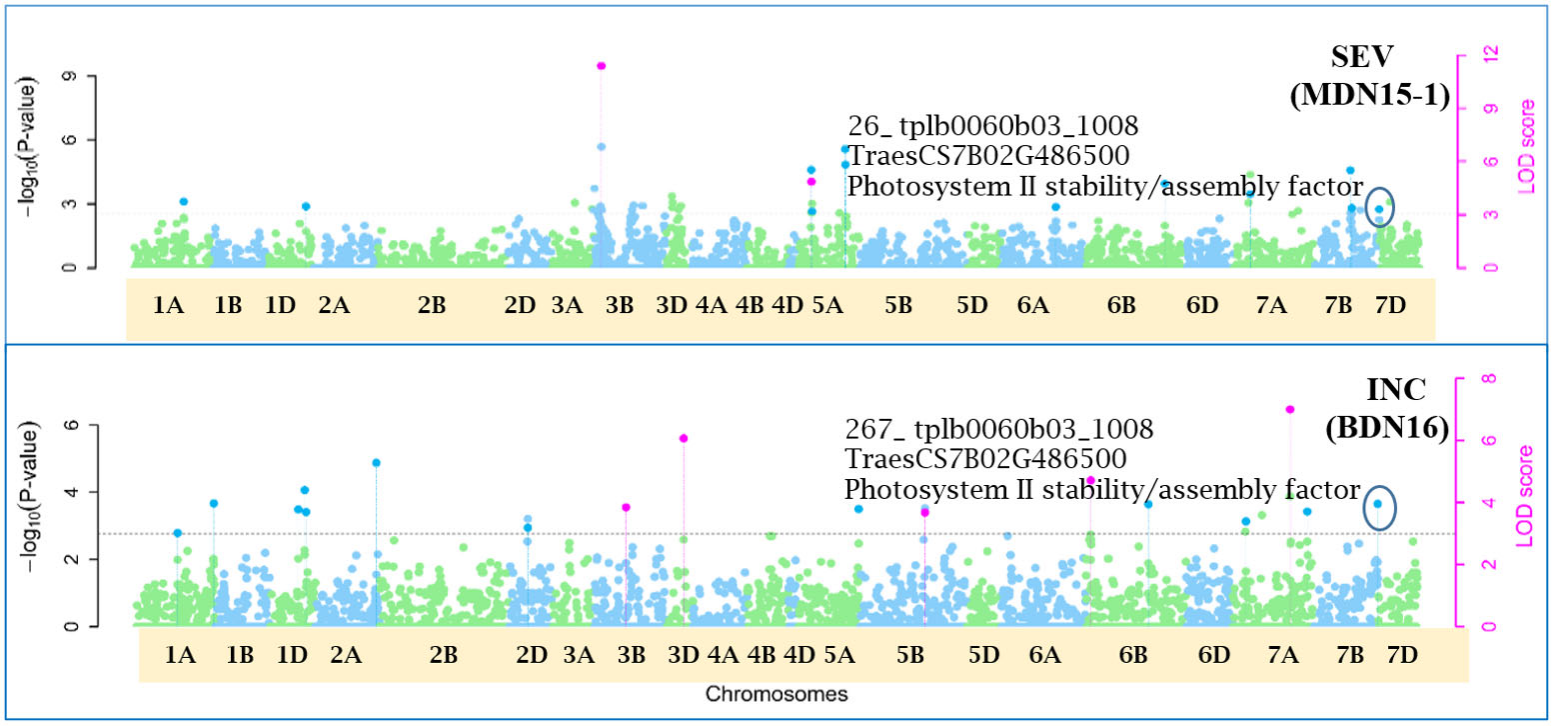
Manhattan plots depicting a pleiotropic Quantitative Trait Nucleotide (QTN) for FHB Incidence (INC) and Severity (SEV), *QTN7B_26-267* on chromosome 7B, detected in MDN 2015 (MDN15-1 dataset) and BDN 2016 environments by the multi-locus random SNP-effect Mixed Linear Model (mrMLM) method deployed on an association mapping panel of 192 predominantly Canadian bread wheats. *QTN7B_26-267* co-locates with a Photosystem II stability/assembly factor (*TraesCS7B02G486500*). The horizontal black dotted line denotes the significance threshold (LOD=3), while pink dots above the threshold line represent QTN detected by more than one of the six multi-locus methods.

Of the 12 pleiotropic QTNs identified in this study, seven were detected for FHB traits, three for FHB INC and DTA, and two for FHB traits and PLHT. Candidate genes associated with each of these QTNs were obtained through a BLASTn search on the online IWGSC RefSeq v2.1 database. The physical locations of QTNs and their associated candidate genes, on all 21 wheat chromosomes, are depicted in Manhattan plots generated *via* the multi-locus mrMLM method (**Figures 5**–**10** and **Supplementary Figures 3**-**5**).

### Pleiotropic QTN for FHB traits

Seven of the 12 QTNs were pleiotropic for FHB INC, SEV, and IND (**Figures 5**–**10**). The first QTN, *QTN3B_9-16-32-77-89-96-164-185*, is located within the *Fhb1* region on chromosome 3B, is associated with SNP marker *CAP7_c1576_371* (positioned at 10.71 Mb), and co-locates with *TraesCS3B02G024900*, which encodes a DEAD/DEAH box RNA helicase domain (3B:15817353..15827081) in RefSeq v2.1 (**Figure 5**). The second pleiotropic QTN for FHB INC, SEV, and IND, *QTN2D_7-31-162-184* is located at 62.96 Mb on chromosome 2D and co-locates with a ribosome biogenesis protein NSA2 homolog gene (2D:65431554..65434070) in RefSeq v2.1 (**Figure 6**). The third pleiotropic QTN (FHB INC, and IND) on chromosome 5A coincides with a beta-amylase protein gene (5A:710066497..710070348) in RefSeq v2.1 (**Figure 7**). The fourth pleiotropic QTN for FHB SEV and IND on chromosome 6BS, *QTN6B_92-106-272*, coincides with a *S*-adenosylmethionine synthase protein (6B:240608170..240610672) in RefSeq v2.1 (**Figure 8**). This QTN is located within the *Fhb2* region (224.1–233.3 Mb; RefSeq v1.1) of chromosome 6B (Zhu et al., 2021). The fifth and sixth pleiotropic FHB trait QTNs on chromosome 7A correspond to a multiple organellar RNA editing factor 2 gene (7A:24246669..24250241; RefSeq v2.1) and an actin–fragmin kinase catalytic domain-containing protein gene (7A:717563958..717569590) in RefSeq v2.1 (**Figure 9**). Lastly, the seventh pleiotropic QTN (FHB SEV and IND) on chromosome 7B, *QTN7B_26-267*, co-locates with a Photosystem II stability/assembly factor (7B:754941324..754943778) in RefSeq v2.1 (**Figure 10**).

### Pleiotropic QTN for FHB INC and DTA

Three QTNs were pleiotropic for FHB INC and DTA. The first on chromosome 1A, *QTN1A_46-252*, co-locates with an uncharacterized protein gene (1A:510051474..510054986) in RefSeq v2.1. The second, *QTN1D_49-255*, on chromosome 1D co-locates with a pentatricopeptide repeat-containing protein (1D:240672206..240673769) in RefSeq v2.1. The third FHB INC and DTA QTN on chromosome 6B, *QTN6B_59-262*, co-locates with an ATP synthase mitochondrial F1 complex assembly factor 1 (6B:567042315..567046184) in RefSeq v2.1 (**Supplementary Figure 3**).

### Pleiotropic QTN for FHB traits and PLHT

Two pleiotropic QTNs for FHB traits and PLHT were detected. The first for FHB SEV, IND, and PLHT, *QTN6B_70-169-194*, located on chromosome 6B, is represented by SNP marker *BS00104265_51* (645.66 Mb) and co-locates with a protein-coding gene (6B:653923276..653926026) in RefSeq v2.0 (**Supplementary Figure 4**). The second QTN for FHB INC, SEV, IND, and PLHT, *QTN7B_13-45-83-94-108-154-176-201-276-291*, on chromosome 7B coincides with MMS19 nucleotide excision repair protein (7B:677195719..677206408) in RefSeq v2.0 (**Supplementary Figure 5**).

### Non-pleiotropic QTN appearing in two or more environments

#### Non-pleiotropic QTN for DTA

Two QTNs for DTA were detected from the MDN 2015 and 2017 environments. The first DTA QTN on chromosome 1B, *QTN1B_48-206* (located at 563.67 Mb), explained 8.28%–46.28% of the phenotypic variance in DTA and co-locates with a disease resistance protein (1B:570333804..570337336) in RefSeq v2.1. The second QTN, *QTN7A_61-219*, on chromosome 7A (located at 675.23 Mb) explained 3.41%–13.96% of the phenotypic variance for DTA and co-locates with an uncharacterized protein (7A:679827145..679827600) in RefSeq v2.1 (**Supplementary Figure 6**).

#### Non-pleiotropic QTN for PLHT

For PLHT, three QTNs were detected in three environments (MDN 2015, MDN 2016, and MDN 2017), two of which were located on chromosome 4B and one on chromosome 6B. The first PLHT QTN on chromosome 4B, *QTN4B_144-226*, is located at 38.28 Mb and accounts for 17.8%–33.9% of the phenotypic variance in PLHT. This QTN is associated with a protein kinase domain-containing protein (4B:41016487..41020890) in RefSeq v2.1. The second 4B QTN, *QTN4B_65-145-227*, (located at 578.04 Mb), explained 3.94%–34.1% of the phenotypic variance and co-locates with a *N*-acetyltransferase domain-containing protein (4B:577090863..577094213) in RefSeq v2.1. The third PLHT QTN on chromosome 6B, *QTN6B_151-229* (located at 8.78 Mb), accounted for 7.66%–43.58% of the phenotypic variance for PLHT and co-locates with an uncharacterized protein (6B:10687959..10689500) in RefSeq v2.1 (**Supplementary Figure 7**).

## Discussion

This study detected pleiotropic QTNs for FHB resistance traits, DTA, and PLHT, as well as single-trait QTN (DTA and PLHT), for the characterization of FHB traits in relation to DTA and PLHT. Overall, highly significant and moderate-to-strong correlations were observed among FHB traits, which however shared weak inverse correlations with PLHT and with DTA. Broad-sense heritability (*H*^2^) values were lower for FHB traits when compared to PLHT and DTA. Multi-locus mrMLM detected a total of 291 statistically significant QTNs, 17 of which were detected at two or more environments of MDN and BDN. Twelve of these 17 QTNs were pleiotropic for INC, SEV, IND, DTA, or PLHT; two QTNs corresponded to DTA and three to PLHT. Consistency of the above results was backed by a separate combined six-environment multi-locus GWAS analyses. Among notable findings of this study are the detection of a pleiotropic QTN within the *Fhb1* region on chromosome 3B, which is ~3 Mb from a cloned *Fhb1* candidate gene *TaHRC*; a putatively novel PLHT QTN on chromosome 6B; a 1B DTA QTN located ~10 Mb from a *Flowering Locus T1-like* gene *TaFT3-B1*; and a DTA QTN on chromosome 7A, which is ~5 Mb from a maturity QTL *QMat.dms-7A.3* of another study. Further, four of the 12 pleiotropic QTNs on chromosomes 1A, 1B, 3B, and 6B are potentially identical to corresponding QTLs in durum wheat. Upon validation of the above QTNs, their suitability for the downstream development of trait-specific markers for breeding selection will be assessed.

The identification of reliable and closely linked markers for MAS of quantitative traits like FHB (Steiner et al., 2017; Sari et al., 2020) is an important step in the development of FHB-resistant wheat cultivars. Several previous studies have deployed MAS for the detection, introgression, and stacking of FHB resistances, for example, the detection of a *Qfhs.ifa-5A* QTL on chromosome 5A (Buerstmayr et al., 2003), and introgression and stacking of FHB resistance QTL (Somers et al., 2005; Miedaner et al., 2006). Studies such as these have led to the development of diagnostic markers like UMN10 for *Fhb1* (Liu et al., 2008; Schweiger et al., 2016). The following paragraphs discuss the significance of the identified FHB, DTA, and PLHT QTNs and their corresponding candidate genes in relation to pertinent findings from previous studies.

### Pleiotropic QTN for FHB traits

Of the 12 pleiotropic QTNs detected in our study, seven were pleiotropic for FHB INC, SEV, and IND. The first of seven, *QTN3B_9-16-32-77-89-96-164-185*, for FHB INC, SEV, and IND, located at 10.71 Mb and within the *Fhb1* region (7.6–13.9 Mb; Wu et al., 2019) on chromosome 3B, is positioned ~1 Mb away from a 3B.2 QTL (9.8 Mb) identified in a durum wheat GWAS by Ruan et al. (2020). This QTN coincides with a DEAD/DEAH box RNA helicase domain (Lasko et al., 1989) and is ~3 Mb away from a cloned *Fhb1* candidate gene *TaHRC* (*T. aestivum* haplotype Clark histidine-rich calcium-binding protein; Su et al., 2019). The DEAD/DEAH box RNA helicase is involved in biotic and abiotic stress responses in wheat (Zhang et al., 2014; Hu et al., 2020), rice (Macovei et al., 2012), *Arabidopsis* (Kim et al., 2008), and tomato (Pandey et al., 2019). Zhang et al. (2014) cloned the wheat DEAD/DEAH box RNA helicase gene *TaRH1* and reported it to be a positive regulator during the defense response to the stripe rust fungus *Puccinia striiformis* f. sp. *tritici* (*Pst*). Further, in a protein–protein interaction study, the DEAD/DEAH box RNA helicase protein *PRH75*, also located on chromosome 5BL, was identified as a key hub protein that is induced in response to the powdery mildew fungus *Blumeria graminis* f. sp. *tritici* (*Bgt*). Given its co-location with FHB trait *QTN3B_9-16-32-77-89-96-164-185* and proximity to *TaHRC* gene (Su et al., 2019) within the *Fhb1* region on chromosome 3B, *TraesCS3B02G024900*, which encodes a DEAD/DEAH box RNA helicase domain, could potentially be a candidate for the *Fhb1* locus. The second pleiotropic *QTN2D_7-31-162-184* for FHB INC, SEV, and IND, represented by SNP marker *wsnp_Ex_c6400_11123059* (located at 62.96 Mb) on chromosome 2D, co-locates with a ribosome biogenesis protein NSA2 homolog (*TraesCS2D02G113600*). In a consensus genetic map by Bokore et al. (2020), SNP marker *wsnp_Ex_c6400_11123059* representing the above QTN is located ~6 cM from SSR marker *Xgwm484*, which, along with marker *Xgwm261*, flanks the photoperiod sensitive gene *Ppd-D1* on chromosome 2D (Gasperini et al., 2012). This genomic region on chromosome 2D also coincides with an anthesis date QTL *QAnth.crc-2D* mapped at ~37.1 cM in a Kenyon/86ISMN recombinant inbred line (RIL) population, which is likely the effect of *Ppd-D1* (McCartney et al., 2016), and *QLr.spa-2D.2*, a QTL associated with leaf rust resistance in Canada and New Zealand, detected in a Stettler/Red Fife spring wheat population (Bokore et al., 2020).

The third pleiotropic *QTN5A_80-102-130-260* for FHB INC and IND on chromosome 5A coincides with a beta-amylase protein gene. Beta-amylase activity is said to increase 10 days after anthesis (LaBerge and Marchylo, 1986). Its expression was reported to be upregulated in response to *Fusarium culmorum* and *F. graminearum* infection of emmer wheat grains (Eggert et al., 2011). The fourth *QTN6B_92-106-272* for FHB SEV and IND co-locates with a *S*-adenosylmethionine (SAM) synthase protein located (224.1–233.3 Mb) within the *Fhb2* region on chromosome 6B (Zhu et al., 2021). In response to inoculation with *F. graminearum*, the SAM protein was upregulated in resistant near-isogenic lines (NILs) carrying the *Fhb1* locus and derived from an HC374/98B69*L47 cross (Gunnaiah et al., 2012). Further, a related SAM-dependent methyltransferase (TaSAM) protein was differentially expressed in microarray analyses comparing transcript accumulation in DON treated versus untreated lines derived from a CM82036/Remus cross, segregating for *Fhb1* (Walter et al., 2008).

The fifth (*QTN7A_40-274-288-173-197*) pleiotropic FHB trait QTN on chromosome 7A corresponds to a multiple organellar RNA editing factor (MORF) 2 gene. MORF family proteins are involved in RNA editing (Benne et al., 1986) in the organelles of flowering plants (Takenaka et al., 2012). The first study of RNA editing or RNA/DNA difference (RDDs) in wheat, in response to *F. graminearum* infection, was carried out by Yang et al. (2022), using publicly available RNA-seq samples of four wheat genotypes, Nyubai, Wuhan 1, HC374, and Shaw. The sixth (*QTN7A_200-266-273-287*) pleiotropic FHB trait QTN, also on chromosome 7A, co-locates with an actin–fragmin kinase (AFK) catalytic domain-containing protein. AFK, an actin-binding protein kinase (Eichinger et al., 1996), along with actins and microtubules, is associated with the plant cytoskeleton (Staiger and Schliwa, 1987; Etienne-Manneville, 2004) and is most likely involved in biotic resistance mechanisms (Kobayashi et al., 1992; Takemoto et al., 2003; Takemoto and Hardham, 2004; Hardham, 2013).

Lastly, the seventh pleiotropic QTN (FHB SEV and IND) on chromosome 7B, *QTN7B_26-267*, co-locates with a Photosystem II (PSII) stability/assembly factor. PSII is bound by oxygen-evolving enhancer proteins (OEEs; Sugihara et al., 2000), which are essential for its oxygen-evolving activity and stability (Mizobuchi and Yamamoto, 1989) during abiotic and/or biotic stresses. Two of these proteins (OEE1 and OEE2) were upregulated in response to FHB inoculation of FHB-resistant wheat cultivar Wangshuibai and an FHB-resistant NIL derived from a Ning 7840/Clark backcross (Wang et al., 2005; Zhang et al., 2013).

### Pleiotropic QTN for FHB INC and DTA

Three QTNs were pleiotropic for FHB INC and DTA. The first on chromosome 1A, *QTN1A_46-252* (508.5 Mb), co-locates with an uncharacterized protein; in addition, it coincides with a durum wheat INC and SEV 1A.3 QTL interval (503–580 Mb) identified by Ruan et al. (2020). The second, *QTN1D_49-255*, on chromosome 1D co-locates with a pentatricopeptide repeat (PPR)-containing protein. PPR proteins (Aubourg et al., 2000; Small and Peeters, 2000) are required for the expression of several organellar genes mainly in the mitochondria or chloroplasts, where they modulate gene expression at the RNA level (Colcombet et al., 2013; Barkan and Small, 2014; Manna, 2015).

Further, the third, *QTN6B_59-262* for FHB INC and DTA QTN on chromosome 6B, co-locates with an ATP synthase mitochondrial F1 complex assembly factor 1 protein, which most likely plays a role in the regulation of oxidative stress. Oxidative stress is reported to be induced in response to *F. graminearum* infection in wheat (Zhou et al., 2005; Golkari et al., 2007). Wheat mitochondrial phosphate transporter (*MPT*) encoding genes catalyze the oxidative phosphorylation of ADP to ATP (Takabatake et al., 1999) and are involved in the modulation of reactive oxygen species (ROS) and regulation of oxidative stress responses (Walter et al., 2008).

### Pleiotropic QTN for FHB traits and PLHT

Among the two QTNs pleiotropic for FHB traits and PLHT, *QTN6B_70-169-194* for SEV, IND, and PLHT on chromosome 6B co-locates with a protein-coding gene, inside a 585–707-Mb interval of a 6B.1 QTL for FHB resistance detected by Ruan et al. (2020) in a durum wheat panel. The second QTN for INC, SEV, IND, and PLHT, *QTN7B_13-45-83-94-108-154-176-201-277-292* (670.3 Mb) on chromosome 7B, coincides with an MMS19 nucleotide excision repair protein. MMS proteins, among other genes, are involved in the excision repair of DNA damaged by ultraviolet (UV) light in plants (Witkin, 1969; Prakash and Prakash, 1977). As all of the above 12 pleiotropic QTN loci for FHB traits, DTA, and PLHT were detected in a globally diverse bread wheat panel, they could potentially be suitable for introgression into elite commercial lines with low FHB resistance, later maturity, or taller PLHT.

### Non-pleiotropic QTN for DTA

Two QTNs for DTA were detected on chromosomes 1B and 7A from the MDN15 and MDN17 datasets. The QTN on chromosome 1B, *QTN1B_48-206* (located at 563.67 Mb), co-locates with a disease resistance protein (570.33–570.337 Mb). It is ~8 Mb from a wheat FLOWERING LOCUS T 1-like gene (*TaFT3-B1*; Zikhali et al., 2017) positioned at 581.4 Mb (Luján Basile et al., 2019; Zhang et al., 2022) and falls within a 1B.1 QTL interval (544–581 Mb) region influencing DTA, PLHT, and FHB traits in durum wheat (Ruan et al., 2020). The second QTN on chromosome 7A, *QTN7A_61-219* (675.23 Mb), co-locates with an uncharacterized protein located ~0.85 Mb (as per RefSeq v2.1) away from a maturity QTL *QMat.dms-7A.3* (680.67–717.91 Mb in RefSeq v2.1) identified in a RIL population of a ‘Peace’ × ‘CDC Stanley’ cross (Semagn et al., 2021). The two DTA QTNs of our study are independent of those detected for FHB resistance traits. For example, the breeder could consider combining the identified FHB QTN loci with DTA loci, in a configuration that provides FHB resistance with desired maturity.

### Non-pleiotropic QTN for PLHT

For PLHT, none of the QTNs detected in our study co-located with any of the commonly deployed reduced height (*Rht*) genes *Rht-B1*, *Rht-D1*, and *Rht-8*. Of the three QTN detected for PLHT, two were located on chromosome 4B, and one was located on chromosome 6B. The first on chromosome 4B, *QTN4B_144-226* (located at 38.28 Mb), is associated with a protein kinase domain-containing protein, located at 10.15 Mb from the *Rht-B1* the gene *TraesCS4B02G043100* (4B:30861268..30863723 bp in RefSeq v2.1) represented by SNP *Tdurum_contig27834_923* (30.86 Mb). Plant protein kinases catalyze the phosphorylation of proteins, some of which are associated with plant growth and development, disease resistance, and abiotic stresses (Tena et al., 2001; Nakagami et al., 2005). For example, in rice, *OsMAPKKK5*, a mitogen-activated protein kinase is reported to be a positive regulator of plant height and yield (Liu et al., 2019).

The second 4B QTN, *QTN4B_65-145-227* (578.04 Mb), is associated with a *N*-acetyltransferase (NAT) domain-containing protein located within a ~30 Mb genomic region (569.2–599.6 Mb in RefSeq v2.1), which harbors genes regulating maturity and flower development (Semagn et al., 2021). NAT proteins are involved in the biosynthesis of melanin, which regulates plant growth and development (Murch et al., 2001; Murch and Saxena, 2002; Arnao and Hernández-Ruiz, 2014).

The third PLHT QTN on chromosome 6BS, *QTN6B_151-229* (located at 8.78 Mb), is associated with an uncharacterized protein. High R^2^ values and LOD scores observed for *QTN4B_65-145-227* (R^2 ^= 17.8–33.96; LOD = 7.58–15.5) and *QTN6B_151-229* (R^2 ^= 7.6–43.58; LOD = 4.18–12.6) suggest that both QTNs could have a significant influence on PLHT. Several GWAS and QTL studies have reported physical locations of various PLHT QTLs and SNP markers on chromosome 6B (Li et al., 2018; Liu et al., 2018; Li et al., 2019; Luján Basile et al., 2019; Malik et al., 2019; Muhammad et al., 2021; Liu et al., 2022). The fact that the physical location of PLHT *QTN6B_151-229* (8.78 Mb) on chromosome 6B of our study does not coincide with the physical locations of 6B QTL loci reported in the above studies suggests the novelty of the *QTN6B_151-229* locus. Therefore, similar to FHB and DTA, breeders might consider recombining the above PLHT QTN and FHB QTN loci in elite lines that lack desirable FHB and PLHT traits and select for FHB-resistant lines with desired plant height and maturity suited to their respective geographical regions. For example, in Canada, recombining FHB resistance with PLHT and DTA would be most desirable in terms of developing FHB-resistant cultivars that are early maturing and semi-dwarf/short stature, making them less prone to lodging and hence beneficial from a yield standpoint. Taller lines have the added advantage of some level of escape from soil residue-borne FHB infection or Type I resistance (Mesterhazy, 1995; Miedaner, 1997; Hilton et al., 1999; Ma et al., 2000; Kolb et al., 2001; Steiner et al., 2004; Srinivasachary et al., 2008; Srinivasachary et al., 2009; Mao et al., 2010; Buerstmayr and Buerstmayr, 2015 and Buerstmayr and Buerstmayr, 2016). However, given the artificial soil inoculation with *F. graminearum* isolates at our nurseries, it would be necessary to determine if the identified pleiotropic (FHB and PLHT) and PLHT QTN are the results of true FHB resistance or disease escape/passive resistance of the taller lines (Mesterhazy, 1995; Klahr et al., 2007).

The detection of *Fhb1* and *Fhb2* would be expected given the presence of Sumai 3 in the AMP, alongside its Canadian derivatives (AAC Brandon, AAC Elie, Cardale, and AC Carberry), all originating from crosses of bridging parents Alsen and/or ND744 (Zhu et al., 2019). In addition to the statistically significant QTNs on chromosomes 1A, 1D, 2D, 3B, 5A, 6B, 7A, and 7B, detected from two or more environments, several single-environment QTNs explaining high phenotypic variances (i.e., R^2^ up to 36%) in INC, SEV, IND, DTA, or PLHT traits were detected. For example, 18 statistically significant QTNs for FHB traits were detected only in the OWA 2017 environment and not in the other two environments of MDN or BDN (**Supplementary Material 3**). All of these single-location QTNs have not been discussed here due to a lack of association with the main 17 QTNs of this study. However, genomic information on these QTNs will be useful for comparative mapping in other bread and durum wheat GWAS, in addition to bi-parental segregating population studies involving parental genotypes, which comprise our AMP.

Another interesting finding of this study might suggest the presence of potentially similar or identical QTLs for FHB, DTA, and/or other traits, in bread and durum wheat, given their proximal or near-identical genomic locations. Based on the results of a durum GWAS by Ruan et al. (2020), each of the four QTNs on chromosomes 1A, 1B, 3B, and 6B of this study shares a common location with their corresponding QTLs in durum wheat. These include *QTN1A_46-252* (508.5 Mb) for INC and DTA on chromosome 1A, whose location coincides with an INC and SEV 1A.3 QTL interval (503–580 Mb) on chromosome 1A of durum wheat. The second, *QTN1B_48-206* (563.67 Mb), for DTA falls within a 1B.1 QTL interval (544–581 Mb) influencing DTA, PLHT, and FHB traits in durum wheat and is located ~8 Mb from a wheat FLOWERING LOCUS T 1-like gene (*TaFT3-B1*). The third, *QTN3B_9-16-32-77-89-96-164-185* (10.71 Mb), for FHB (INC, SEV, and IND), which was detected within the *Fhb1* region on chromosome 3B, is located in close proximity to a 3B.2 (9.8 Mb) QTL of durum wheat. Finally, the fourth pleiotropic *QTN6B_70-169-194* for SEV, IND, and PLHT on chromosome 6B of this study is located within a 585–707-Mb interval of a 6B.1 QTL for FHB resistance in durum wheat.

## Conclusion

Given that FHB is a yield and quality-limiting disease of wheat, there is a constant need for reliable SNP markers to identify FHB-resistant genotypes. The 17 QTNs detected in this study are potentially significant from a breeding and cultivar improvement perspective. Among notable ones are the 3B QTN within the *Fhb1* region, a 1B DTA QTN close to a Flowering Locus T1-like gene TaFT3-B1, a putatively novel 6B PLHT QTN, a 7A DTA QTN close to a maturity QTL *QMat.dms-7A.3* of another study, and four pleiotropic QTNs, all potentially identical to their counterparts in durum wheat. Further assessment and validation of the identified QTNs are merited, for example, in bi-parental segregating or doubled-haploid populations tested across multiple environments. Once validated, the above QTN-derived KASP markers would provide breeders with options to deploy pleiotropic loci for FHB resistance and PLHT or recombine FHB resistance with PLHT and/or with DTA loci and select for FHB-resistant lines with desired maturity and height.

## Data availability statement

The original contributions presented in the study are included in the article/**Supplementary Material**. Further inquiries can be directed to the corresponding authors.

## Author contributions

RK, YR, and RC conceived, designed, and provided oversight on the study. RC and RK constructed the bread association mapping (AM) population. RC, RK, and YR contributed to the bread germplasm development and seed increase in the AM population. MH, SK, AB, SB, FB, IP, YR, RC, and RK performed field trials and disease evaluation at FHB nurseries located in Morden and Brandon, Manitoba, and Ottawa, Ontario. WZ, KB, and PF contributed to the genotyping. BM, WZ, and LL generated an initial genetic map. AC performed all statistical analyses as part of the GWAS for QTNs and candidate detection and prepared the original draft. AC, RK, YR, and RC analyzed the data and interpreted the results. AC, RK, and YR edited the original draft. All authors contributed to the article and approved the submitted version.
