# Supplementary material for: Multi-locus genome-wide association study of fusarium head blight in relation to days to anthesis and plant height in a spring wheat association panel
Source: Front Plant Sci. 2023 Jun 29;14:1166282. doi: 10.3389/fpls.2023.1166282 (PMC10346453; doi:10.3389/fpls.2023.1166282)
Supplement: Supplementary file 2 [file DataSheet_2.pdf]

Supplementary Figure 2

Article title:

Multi-locus Genome-wide Association Study of Fusarium Head Blight in relation to Days to Anthesis and Plant Height in a Spring Wheat Association Panel

Authors:

Adrian L. Cabral[1], Yuefeng Ruan[1*], Richard Cuthbert[1*], Lin Li[1], Wentao Zhang[2], Samia Berraies[1], Maria Antonia Henriquez[3], Andrew Burt[4], Santosh Kumar[5], Pierre R. Fobert[6], Isabelle Piche[1], Firdissa Bokore[1], Brad Meyer[1], Jatinder Sangha[1], Ron Knox[1]

[1]Swift Current Research and Development Centre, Agriculture and Agri-Food Canada, Box 1030, 1 Airport Road, Swift Current, SK S9H 3X2 Canada
[2]Aquatic and Crop Resource Development Research Centre, National Research Council of Canada, 110 Gymnasium Place, Saskatoon, SK, Canada, S7N 0W9
[3]Morden Research and Development Centre, Agriculture and Agri-Food Canada, 101 Route 100, Morden, MB, Canada, R6M 1Y5
[4]Ottawa Research and Development Centre, Agriculture and Agri-Food Canada, Ottawa, ON, K1A 0C6
[5]Brandon Research and Development Centre, Agriculture and Agri-Food Canada, 2701 Grand Valley Road, Brandon, MB, Canada, R7A 5Y3
[6]Aquatic and Crop Resource Development Research Centre, National Research Council of Canada, 100 Sussex Drive, Ottawa, ON, K1N 5A2

Corresponding Author affiliation and email address :

[1]Swift Current Research and Development Centre, Agriculture and Agri-Food Canada, Box 1030, 1 Airport Road, Swift Current, SK S9H 3X2 Canada
yuefeng.ruan@agr.gc.ca
richard.cuthbert@agr.gc.ca

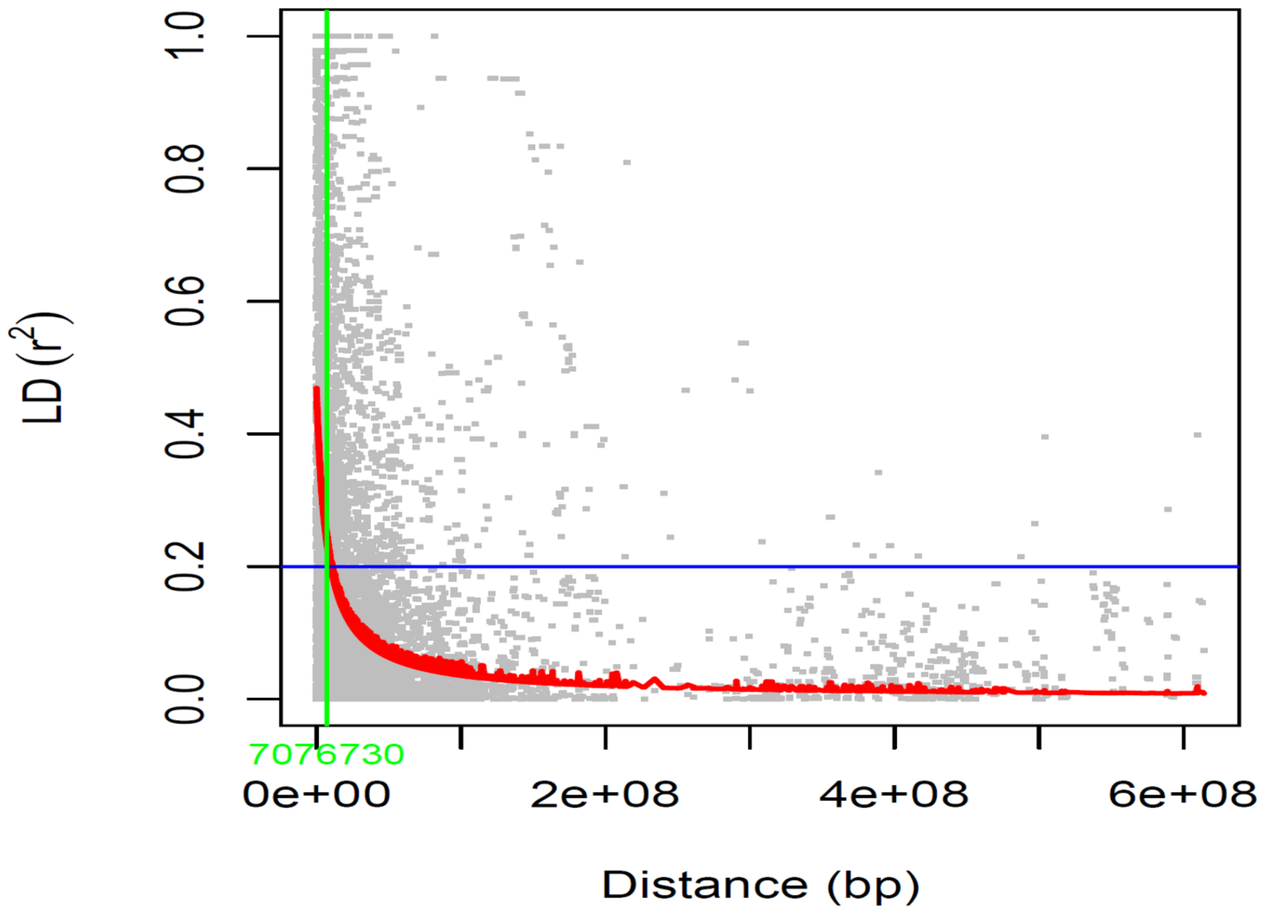

Supplementary Figure 2. A genome-wide Linkage disequilibrium (LD) scatter plot depicting LD (r^2) of marker-pairs versus physical distance in base pairs (bp), generated from 5441 SNP markers run on an association mapping panel of 192 predominantly Canadian bread wheat genotypes. LD decay below the 0.2 r^2 threshold occurs at a physical distance of approximately 7.08 Mb, which is the intersection of the blue horizontal threshold line and the LOESS (locally estimated scatterplot smoothing) curve in red
