# Supplementary material for: Multi-locus genome-wide association study of fusarium head blight in relation to days to anthesis and plant height in a spring wheat association panel
Source: Front Plant Sci. 2023 Jun 29;14:1166282. doi: 10.3389/fpls.2023.1166282 (PMC10346453; doi:10.3389/fpls.2023.1166282)
Supplement: Supplementary file 4 [file DataSheet_4.pdf]

Supplementary Figure 4

Article title:

Multi-locus Genome-wide Association Study of Fusarium Head Blight in relation to Days to Anthesis and Plant Height in a Spring Wheat Association Panel

Authors:

Adrian L. Cabral[1], Yuefeng Ruan[1*], Richard Cuthbert[1*], Lin Li[1], Wentao Zhang[2], Samia Berraies[1], Maria Antonia Henriquez[3], Andrew Burt[4], Santosh Kumar[5], Pierre R. Fobert[6], Isabelle Piche[1], Firdissa Bokore[1], Brad Meyer[1], Jatinder Sangha[1], Ron Knox[1]

[1]Swift Current Research and Development Centre, Agriculture and Agri-Food Canada, Box 1030, 1 Airport Road, Swift Current, SK S9H 3X2 Canada
[2]Aquatic and Crop Resource Development Research Centre, National Research Council of Canada, 110 Gymnasium Place, Saskatoon, SK, Canada, S7N 0W9
[3]Morden Research and Development Centre, Agriculture and Agri-Food Canada, 101 Route 100, Morden, MB, Canada, R6M 1Y5
[4]Ottawa Research and Development Centre, Agriculture and Agri-Food Canada, Ottawa, ON, K1A 0C6
[5]Brandon Research and Development Centre, Agriculture and Agri-Food Canada, 2701 Grand Valley Road, Brandon, MB, Canada, R7A 5Y3
[6]Aquatic and Crop Resource Development Research Centre, National Research Council of Canada, 100 Sussex Drive, Ottawa, ON, K1N 5A2

Corresponding Author affiliation and email address :

[1]Swift Current Research and Development Centre, Agriculture and Agri-Food Canada, Box 1030, 1 Airport Road, Swift Current, SK S9H 3X2 Canada
yuefeng.ruan@agr.gc.ca
richard.cuthbert@agr.gc.ca

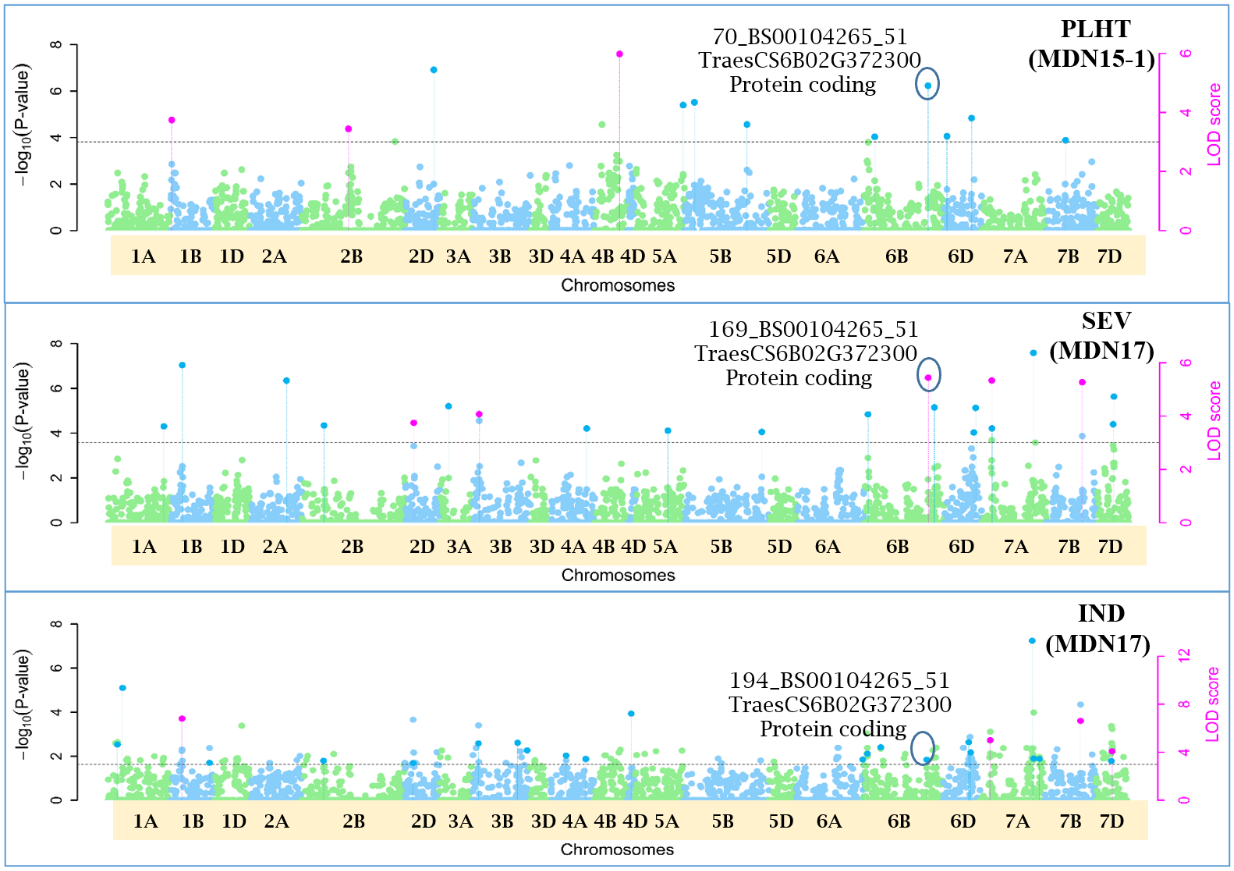

Supplementary Figure 4. Manhattan plots depicting a pleiotropic Quantitative Trait Nucleotide (QTN), *QTN6B_70-169-194* for Plant Height (PLHT), FHB Severity (SEV) and Index (IND) on chromosome 6B, represented by SNP marker *BS00104265_51*, coinciding with a protein coding gene (*TraesCS6B02G372300*), detected from Morden 2015 (MDN15-1 dataset) (*top*) and Morden 2017 (MDN17 datasets) environments (*middle and bottom plots*) by the multi-locus random SNP-effect Mixed Linear Model (MrMLM) method deployed on an association mapping panel of 192 predominantly Canadian bread wheats. The horizontal black dotted line denotes the significance threshold (LOD=3), while pink dots above the threshold line represent QTN detected by more than one of the six multi-locus methods
